# Cell-only bioprinting of articular cartilage progenitor cells within a physically constraining support bath to engineer structurally organized grafts

**DOI:** 10.1016/j.bioactmat.2025.12.013

**Published:** 2025-12-29

**Authors:** Aliaa S. Karam, Gabriela S. Kronemberger, Kaoutar Chattahy, Daniel J. Kelly

**Affiliations:** a Trinity Centre for Biomedical Engineering, Trinity Biomedical Sciences Institute, Trinity College Dublin, Dublin, D02 PN40, Ireland; b Department of Mechanical, Manufacturing and Biomedical Engineering, School of Engineering, Trinity College Dublin, Dublin, Ireland; c Advanced Materials and Bioengineering Research Centre (AMBER), Royal College of Surgeons in Ireland and Trinity College Dublin, Dublin, Ireland; d Department of Anatomy and Regenerative Medicine, Royal College of Surgeons in Ireland, Dublin, Ireland

**Keywords:** Articular cartilage, Embedded bioprinting, Collagen alignment, Boundary conditions, Methacrylated xanthan gum

## Abstract

Engineering functional articular cartilage (AC) grafts is one of the greatest challenges in tissue engineering. Recapitulating the arcade-like collagen organisation of AC, which is integral to the tissues’ strength and stiffness, is necessary to engineer truly functional grafts. This motivates the need for innovative strategies to control collagen alignment in engineered tissues in a programmable manner. Emerging 3D bioprinting strategies can provide spatially defined cues to guide tissue growth. Therefore, the goal of this study was to use embedded bioprinting to provide spatially defined boundary conditions to AC progenitor cells (ACP) to direct collagen organization and support the development of biomimetic cartilage tissues. ACPs were isolated through differential adhesion to fibronectin and demonstrated superior chondrogenesis to donor matched chondrocytes. Two different approaches (casting and 3D bioprinting) were used to physically constrain ACPs with external boundaries of differing widths (250, 500, or 750 μm). For both approaches, thinner boundaries promoted greater collagen alignment along the long axis of the developing tissue. Building on this, ACPs were bioprinted into a sheet, with collagen fibers aligning parallel to the print direction. Finally, a multi-layered graft was bioprinted with horizontal filaments (XY plane) overlaying vertical filaments (Z-axis). The bioprinted tissue had an arcade-like collagen organization with horizontal collagen fibres overlaying vertical collagen fibres. These findings demonstrate how support baths can be used to provide spatially defined physical boundary conditions to bioprinted cells to guide matrix organization, enabling the engineering of anisotropic AC grafts.

## Highlights

• Articular cartilage (AC) progenitors are superior to chondrocytes for AC engineering.
• Physical boundaries direct the organization of extracellular matrix secreted by ACPs.
• Greater physical confinement enhances cell and collagen alignment.
• Support baths can provide spatially defined boundaries that direct tissue growth.
• Physical boundaries support the bioprinting of anisotropic cartilage grafts.

## Graphical abstract

A) diagrammatic representation of physical boundaries directing collagen alignment of a confined bioink. The smaller the spacing X between the boundary the greater the neotissue collagen alignment. B) Utilizing an embedded 3D bioprinting support bath as a physical boundary to direct a biomimetic arcade-like collagen alignment in bioprinted anisotropic articular cartilage sheet.

**Figure:**
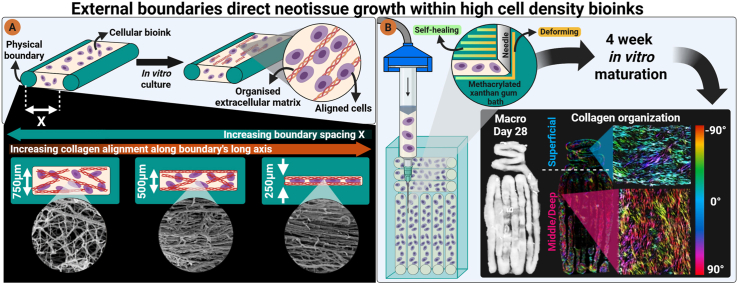

## 1 Introduction

Similar to many tissues in the musculoskeletal system, AC has a highly structured extracellular matrix (ECM) that is integral to its function. Specifically, the unique arcade-like collagen architecture of AC is crucial for its load-bearing properties, allowing it to resist applied stresses and facilitate locomotion [1,2]. Parallel fibrils in the superficial zone are fundamental in resisting high tensile stresses [1], while vertical collagen fibrils in the deep zone anchor the AC to the underlying subchondral bone and provide protection against large strains [2]. AC has a poor self-healing potential and even small lesions, if left untreated, can progress to osteoarthritis [3], a painful and debilitating disease affecting more than 300 million individuals worldwide [4]. Current treatment options for AC lesions include bone marrow stimulation through microfracture of the subchondral bone [5] and autologous chondrocyte implantation [6]. Both treatments form an unorganized fibrocartilage-like repair tissue that eventually fails due to poor mechanical properties that do not satisfy the functional demands of the joint, leaving patients still at a high risk for developing osteoarthritis [7,8]. Alternatively, osteochondral grafting results in a more functional repair with a graft survival rate of more than 80 % after 15 years of implantation [9,10]. However, with allografts there is a limited donor supply, and they can only be used to treat small lesions [11]. Therefore, there is an evident need for novel treatments for biological joint resurfacing.

Engineering functional AC grafts is a challenging goal in the field of cartilage tissue engineering (TE). Realising this goal first requires identifying a suitable cell source capable to generating phenotypically stable AC. The primary cells in AC (the chondrocytes) have a low metabolic activity, however, there does exist a pool of progenitor cells that primarily reside in the superficial zone of the tissue [12]. These AC progenitor cells (ACPs), which were identified over 20 years ago [12], are increasingly being used in the field of cartilage TE [13,14]. In contrast to the widely used mesenchymal stem/stromal cells (MSC) and culture expanded chondrocytes, ACPs have a unique ability to maintain a stable hyaline cartilage phenotype [[15], [16], [17]]. In addition to identifying a suitable cell source, engineering functional AC also requires recapitulating the complex zonal composition and organization of the native tissue. While multilayered scaffolds and hydrogels have been developed to mimic certain aspects of the zonal composition of AC [[18], [19], [20], [21], [22], [23], [24], [25], [26], [27]], they typically fail to produce engineered tissues with an arcade-like collagen network. Computational modelling has shown that achieving this collagen organisation is the most important parameter in determining the functional success of an engineered AC graft [28]. This motivates the need for innovative TE strategies that can direct collagen alignment and, hence, generate functional AC grafts.

During embryonic development different tissues form concurrently, exerting mechanical forces on one another that influence morphogenesis [29]. Similarly, cells *in vitro* can sense their surrounding microenvironment and respond to mechanical, physical, and geometrical cues [30]. Leveraging this, emerging 3D bioprinting techniques could potentially be used to address limitations of classical TE strategies by providing spatially and temporally defined biochemical and biophysical cues to cells as they self-organise into a structurally organised tissue. For example, polycaprolactone (PCL) scaffolds with elongated pores have been show to function as external boundaries that can direct cell and collagen alignment when cells are placed into these biomaterials [31,32]. However, PCL degrades relatively slowly *in vivo* and can promote fibrosis [33,34]. Therefore, there is a need for novel strategies to direct collagen organization in TE AC grafts.

Embedded bioprinting techniques have enabled high resolution printing without the need for high viscosity bioinks [35]. This involves the bioprinting of a low viscosity bioink in a hydrogel support bath which typically possess thixotropic and shear recovery behaviours. Different biomaterials have been explored for embedded bioprinting including xanthan gum (XG) [[36], [37], [38], [39]], an inert FDA approved polysaccharide secreted by the Xanthomonas Campestris bacteria [40]. It forms viscous hydrogels even at low concentrations. It is non-toxic and stable over a wide range of temperatures and pH and, therefore, widely used in the food industry as a thickening agent [41]. XG has also been explored in the treatment of AC lesions. For instance, the intra-articular injection of XG in a rabbit model of OA has demonstrated a protective function, delaying the progression of OA [42]. Similarly, an MSC laden methacrylated XG (XGMA) bioink was used to bioprint scaffolds for cartilage repair and demonstrated a hyaline-like repair tissue in a rabbit cartilage defect model [43]. In 3D bioprinting XG has been used as a sacrificial support bath to enable the fabrication of gravity defying structures [36,37], while XGMA has previously been used as a permanent cell-laden support bath to bioprint 3D structures with perfusable channels [36]. Recently we have used a XGMA support bath to direct the fusion of bioprinted MSC derived microtissues and their re(modelling) into structurally organised tissue grafts [39]. This suggests that such support baths can be used to provide tuneable physical boundary conditions to developing engineered tissues.

The overall aim of this study was to engineer structurally organised AC grafts with an anisotropic collagen architecture by 3D bioprinting high densities of ACPs into a XGMA support bath. To achieve this, we first isolated caprine ACPs and compared their chondrogenic potential to donor matched chondrocytes to confirm their utility as a cell source for this study. We then investigated how the physical constraint provided by external physical boundaries of different sizes (specifically different channel widths; see Fig. 2) influences the growth and organization of tissue deposited by ACPs. Having confirmed the capacity of ACPs to respond to such physical cues, we then utilized a XGMA support bath to impose spatially defined boundary conditions to ACPs to direct neotissue development and ultimately engineer cartilage with a biomimetic, arcade-like, collagen organization.

## 2 Material and methods

### 2.1 Isolation and expansion of ACPs and chondrocytes

ACPs were isolated from full thickness AC shavings of skeletally mature female goats through differential adhesion to fibronectin (Fig. 4). AC shavings were obtained from animals euthanized as part of a separate, ethics-approved project by the Irish Health Products Regulatory Authority (approval number AE18982). Cell culture flasks were coated with 10 μg/mL fibronectin (Brennan & Co) in 0.1 M Dulbecco's phosphate buffer (PBS) with 1 mM magnesium chloride and 1 mM calcium chloride (CaCl_2_) overnight at 4 °C (all Sigma, Ireland) [12]. The AC was rinsed with PBS and then digested in pronase (70 U/mL, ThermoFischer Scientific) for 30 min at 37 °C. Using a scalpel, AC shavings were chopped to 1–2 mm^3^ pieces. The minced AC was then digested in collagenase type II (300 U/mL, Gibco, Ireland) overnight at 37 °C. Enzymes were prepared in Dulbecco's Modified Eagle Medium/Nutrient Mixture F-12 (DMEM/F-12, ThermoFischer Scientific) supplemented with 100 U/mL penicillin, 100 μg/mL streptomycin, and 2.5 μg/mL amphotericin B (all Gibco, Ireland). Next the digested AC was filtered through a 70 μm sieve and centrifuged at 500 RPM for 5 min. Cells were then seeded into the fibronectin coated flasks. After 20 min, non-adherent cells (chondrocytes) were removed and seeded into uncoated flasks. Chondrocytes were cultured in expansion media (XPAN) composed of high-glucose DMEM (Bioscience, Ireland) supplemented with 10 % (v/v) fetal bovine serum (FBS, Gibco, Ireland), 100 U/mL penicillin, 100 μg/mL streptomycin, and 2.5 μg/mL amphotericin B (Sigma, Ireland). The adherent cells (ACPs) were cultured in XPAN-12 composed of DMEM/F-12 supplemented with 10 % (v/v) FBS, 100 U/mL penicillin, 5 ng/mL of FGF-2 (PeproTech, USA), 100 μg/mL streptomycin, and 2.5 μg/mL amphotericin B. On day 2, ACPs were switched to A_XPAN composed of XPAN-12 supplemented with 0.5 μg/mL of L-ascorbic acid 2-phosphate (Sigma, Ireland) and 1 ng/mL of TGF-β1 (PeproTech, USA). Media changes were performed every 2–3 days. At 80 % confluency, chondrocytes and ACPs were harvested with trypsin (Sigma, Ireland) and TrypLE (Biosciences, Ireland), respectively.

### 2.2 Pellet culture

Pellets were formed by centrifuging 250,000 passage 3 ACPs or chondrocytes 5000 RPM for 5 min. Pellets were cultured in chondrogenic media (CDM+) composed of DMEM GlutaMAX supplemented with 100 U/mL penicillin, 100 μg/mL of streptomycin (both Biosciences, Ireland), 100 μg/mL of sodium pyruvate, 40 μg/mL L-proline, 50 μg/mL L-ascorbic acid-2-phosphate, and 1.5 mg/mL of bovine serum albumin (BSA), 50 μg/mL L-ascorbic acid-2-phosphate, 100 nM of dexamethasone, 4.7 μg/mL of linoleic acid (all Sigma, Ireland), 1X insulin-transferrin-selenium (Biosciences, Ireland), and 10 ng/mL of human transforming growth factor-β3 (TGF- β3) (PeproTech, USA) for 21 days. This was performed for caprine ACPs with donor matched chondrocytes for a total of 3 different donors.

### 2.3 Oxidised alginate (OA) preparation

As previously described [44], alginate was oxidised by dissolving 1 g of sodium alginate (Sigma, Ireland) in 90 mL of deionized water overnight under continuous stirring at room temperature. For a theoretical oxidation of 4 %, 10 mL of sodium periodate (Sigma, Ireland) was added and the reaction was allowed to take place for 24 h in the dark at room temperature. The OA was dialysed for 3 days (MWCO 3.5 kDa) and then freeze-dried.

### 2.4 Fabrication of agarose boundaries and bioink casting

Positive moulds with a 12 mm long, 1 mm high, and 250, 500 or 750 μm wide cuboid were designed on Autodesk Fusion 360 and printed with high temperature resin using a Form 2 stereolithography printer (Formlabs, United States). The moulds were UV cured for 30 min at 60 °C and then used to cast cuboidal channels in molten 4 % (w/v) agarose (Fig. 2A). The bioink was prepared by dissolving 4 % (w/v) OA in XPAN at 37 °C. Passage 3 ACPs cell suspension was centrifuged, and the supernatant was carefully removed. The 4 % OA was then mixed with the cell pellet at a density of 80x10^6^ cells/mL OA. The bioink was then cast into the agarose channels and crosslinked with 60 mM CaCl_2_ for 45 min at 37 °C. Next, 2 % (w/v) agarose (50 °C) was cast on top of the bioink to generate an upper agarose boundary. The agarose was allowed to solidify (5 min) before adding media. The casted cuboids were cultured in CDM+ for 21 days.

### 2.5 Methacrylated xanthan gum preparation

XGMA bioprinting support bath was prepared as previously described [36,39]. Briefly, 0.5 g of xanthan gum (Sigma, Ireland) was dissolved in 100 mL distilled water. Afterwards, 4 mL of glycidyl methacrylate (Sigma, Ireland) was added to the XG at 60 °C overnight. The solution was dialysed (MWCO 6–8 kDa) for one week at room temperature in the dark. Subsequently, the solution was freeze dried and stored at −20 °C until use. For bioprinting XGMA (1 % (w/v)) was dissolved in phenol free DMEM supplemented with 100 U/mL penicillin, 100 μg/mL streptomycin, and 2.5 μg/mL amphotericin B. Lithium phenyl-2,4,6-trimethylbenzoylphosphinate (LAP) (Sigma, Ireland) was added as a photoinitiator (0.25 % (w/v)).

### 2.6 3D bioprinting

The bioink was prepared by centrifuging passage 4 ACPs and loading into a syringe with a 25G needle without a supporting ink (cell-only bioprinting). To achieve different filament widths the printing speed (5–15 mm/s) was varied. Post bioprinting the XGMA bath was crosslinked with UV light (intensity of 12 mW/cm^2^) for 4 min. Bioprinted filaments were cultured in CDM+ for 21 days with media changes every 2–3 days. ACPs were bioprinted into a single-layered sheet in the XY plane with horizontal and vertical filaments to achieve a biomimetic AC collagen organization and cultured for 28 days in CDM+. Afterwards a two-layered graft (either with or without internal boundaries by adjusting print speed) was bioprinted with horizontal filaments in the XY plane overlaying vertical filaments in the Z-axis and also cultured for 28 days in CDM+ with media changes every 2–3 days. Prior to histological and biochemical assays XGMA was removed from bioprinted samples through careful mechanical disruption using a scalpel and tweezer.

### 2.7 LIVE/DEAD imaging

Cell viability was evaluated using a Live/Dead© assay kit (Invitrogen, Bioscience). Bioprinted constructs were washed twice with PBS. A staining solution containing 2 μM calcein and 4 μM ethidium homodimer-1 (EthD-1) was prepared. Samples were incubated at 37 °C for 1 h in the staining solution. After incubation, the constructs were washed twice with PBS then imaged immediately using a Leica SP8 scanning confocal microscope. Calcein was excited at 485 nm with emission detected at 530 nm, while EthD-1 was excited at 530 nm with emission collected at 645 nm. Maximum intensity z-stack projections were generated to assess cell viability throughout the full depth of the construct.

### 2.8 Histological evaluation

Samples were fixed with 4 % paraformaldehyde overnight at 4 °C. Following fixation, samples were embedded in 2 % (w/v) agarose and then dehydrated in increasing concentrations of ethanol solutions (50 %–100 %), cleared in xylene, and embedded in paraffin wax (all Sigma, Ireland). Using a microtome (Leica Microsystems, Ireland) samples were sectioned at 5 μm and then stained with hematoxylin and eosin (H&E), alcian blue (1 % (w/v), pH 1) (AB) to visualise sulphated glycosaminoglycan (sGAG) content and counter-stained with nuclear fast red (0.1 % (w/v)), picrosirius red (PR) (0.1 % (w/v)) for collagen deposition, and alizarin red (1 % (w/v), pH 4.1) for calcium deposition (all from Sigma, Ireland). Stained slides were imaged using an Aperio ScanScope slide scanner. Polarized light microscopy (PLM) was used to image slides stained with PR. On ImageJ collagen fibre orientation and coherency was determined [32]. For each sample PLM quantification analysis was performed on multiple section depths.

### 2.9 Immunohistochemistry analysis

To identify the types of collagens produced, diaminobenzidine (DAB) immunohistochemistry for collagens type I (Abcam ab90395 1:400), type II (Santa Cruz sc52658 1:400), type X (Abcam ab49945 1:200) and lubricin (Millipore MABT400 1:500) were performed as previously described [24,31]. For the detection of hypoxia-inducible factor 1 alpha (HIF-1α), heat antigen retrieval was performed using a sodium citrate buffer (pH = 6) for 20 min in a pressure cooker. Non-specific binding was blocked with 1 % BSA and 10 % donkey serum (Sigma, Ireland) prepared in PBS for 1 h at room temperature. A mouse monoclonal anti-HIF-1α primary antibody (Abcam ab8366 1:200 dilution) was then incubated on the samples overnight at 4 °C. Samples were incubated for 15 min at room temperature in a 1 % hydrogen peroxide (Sigma, Ireland) solution to quench endo-peroxidase activity. Next, slides were incubated with the secondary antibody (Anti-IgG mouse (Sigma B7151, 1:150 dilution) for 1 h at room temperature. Staining was developed using a DAB substrate kit (Vector Labs). Stained slides were imaged using an Aperio ScanScope slide scanner.

### 2.10 Biochemical analysis

Samples were digested in papain (3.88 U/mL) prepared in ultra-pure water containing 0.1 M sodium acetate, 5 mM L-cysteine–hydrochloride hydrate, 5 mM ethylenediaminetetraacetic acid (EDTA) and the pH was adjusted to 6.5 using 1 M hydrochloric acid (HCl) (all from Sigma, Ireland) overnight at 60 °C under continuous rotation (40 RPM). DNA content was quantified after digestion using Quant-iT™ PicoGreen ® dsDNA Reagent and Kit (Molecular Probes, Biosciences). Sulphated glycosaminoglycan (sGAG) content was determined using the 1,9-Dimethyl-Methylene blue (Sigma, Ireland) dye (pH of 1.5), with a chondroitin sulfate (Blyscan, Biocolor Ltd., Carrickfergus, UK) standard. Excitation was measured at 590 and 530 nm using the Synergy HT multi-detection micro-plate reader (BioTek Instruments, Inc). Total collagen content was quantified by measuring the hydroxyproline content using the hydroxyproline-to-collagen ratio of 1:7.69 [45]. Briefly, the digested samples were mixed with 38 % HCl and then incubated for 18 h at 110 °C to allow for hydrolysis of hydroxyproline to occur. Samples were then allowed to dry in a fume hood for 2 days. The sediment was resuspended in ultra-pure water and mixed with 2.82 % (w/v) Chloramine T and 0.05 % (w/v) 4-(Dimethylamino) benzaldehyde (both Sigma). A trans-4-Hydroxy-L-proline (Fluka analytical) standard was used. The plate was read at 570 nm Synergy HT multi-detection micro-plate reader (BioTek Instruments, Inc).

### 2.11 Scanning electron microscopy (SEM)

Samples for SEM were fixed in 3 % (w/v) glutaraldehyde for 2 h at room temperature followed by a sequential enzymatic treatment with chondroitinase ABC (0.6 U/mL, 12 h at 37 °C) and hyaluronidase (20.4 U/mL, 12 h at 37 °C) (all from Sigma). Subsequently, samples were dehydrated with increasing concentrations of ethanol (30 %, 50 %, 70 %, 90 % and 100 % at 10 min each) followed by critical point drying. Hence the samples were carefully placed on SEM pin stubs and sliced along the long axis of the sample to expose the inside. A Cressington 208h sputter coater was used to coat the samples with gold/palladium for 1 min. Samples were imaged with a Zeiss ULTRA plus SEM. The diameter of the collagen fibers was measured using ImageJ software.

### 2.12 RNA isolation and quantitative real-time PCR

On day 7 samples were harvested and washed with 0.5 M sodium citrate and then centrifuged to collect cells. Samples were snap frozen and stored at −80 °C until use. RNA was isolated using the High Pure RNA Isolation Kit (Roche, Switzerland) following the manufacturer's instructions. Next, 500 ng of RNA was transcribed to cDNA using a HighCapacity RNA-to-cDNA™ Kit (Applied Biosystems™) through a polymerase chain reaction (PCR) for 1 h at 37 °C, 5 min at 95 °C and 5 min at 4 °C. Real-time PCR was performed with Taqman PCR Fast-Advanced master mix (ThermoFisher) on an Applied Biosystems instrument. In each well goat specific housekeeping gene 18S rRNA TaqMan probe (ThermoFisher) was added with the goat-specific TaqMan probe target gene (ThermoFisher). The qPCR amplification process comprised of 2 min at 50 °C, 10 min at 95 °C and 40 cycles at 60 °C for 1 min each. Using the 2^(−ΔΔCt)^ method gene expression levels were quantified and normalized to the average expression of the 18S rRNA housekeeping gene and to an ACP monolayer.

### 2.13 Rheological assessment of cell-only bioink

**Fig. S1:**
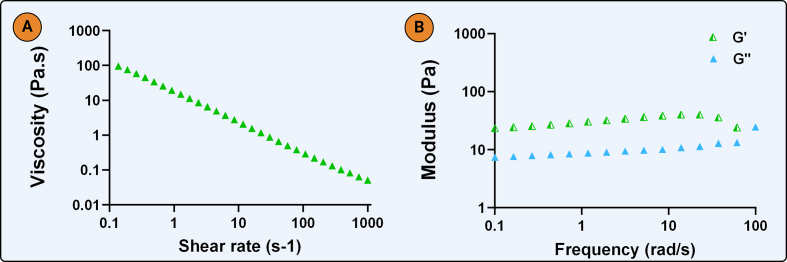
Rheological testing of 1 % (w/v) XGMA support bath demonstrating shear thinning behaviour (n = 3).

Passage 4 ACPs were harvested with TryplE centrifuged and the media was completely removed. The cell pellet at the bottom of the tube is the cell only bioink which is then pipetted with a viscous pipette onto the rheometer (MCR 102, Anton-Paar, Dublin, Ireland) plate with a 25 mm plate diameter (Fig. 4B). The viscosity as a function of shear rate (0.1–1000/s) was measured at room temperature. An amplitude sweep test was conducted at a fixed angular frequency of 1 Hz over 0.01 %–1000 % shear strain at room temperature. Likewise, the rheological properties of 1 % XGMA were also measured (Fig. S1).

### 2.14 Mechanical testing

Following 4 weeks of *in vitro* culture, uniaxial tensile testing of 3D bioprinted sheets was performed on a tensile biaxial tester (CellScale) with a 23 N load cell. Tests were performed at room temperature in a PBS bath. A 0.01 N preload was applied followed by a 10 % strain and held for 10 min until equilibrium was reached. The ramp modulus was determined from the linear phase of the stress-strain curves. The equilibrium modulus was calculated using the equilibrium force.

### 2.15 Statistical analysis

Statistical differences were identified using the unpaired student's t-test or analysis of variance (ANOVA) with Tukey's multiple comparisons using GraphPad Prism software (GraphPad Software, CA, USA). Data was presented as mean ± standard deviation. Significance was accepted when p < 0.05.

## 3 Results and discussion

### 3.1 ACPs are superior to chondrocytes for cartilage tissue engineering

**Fig. 1:**
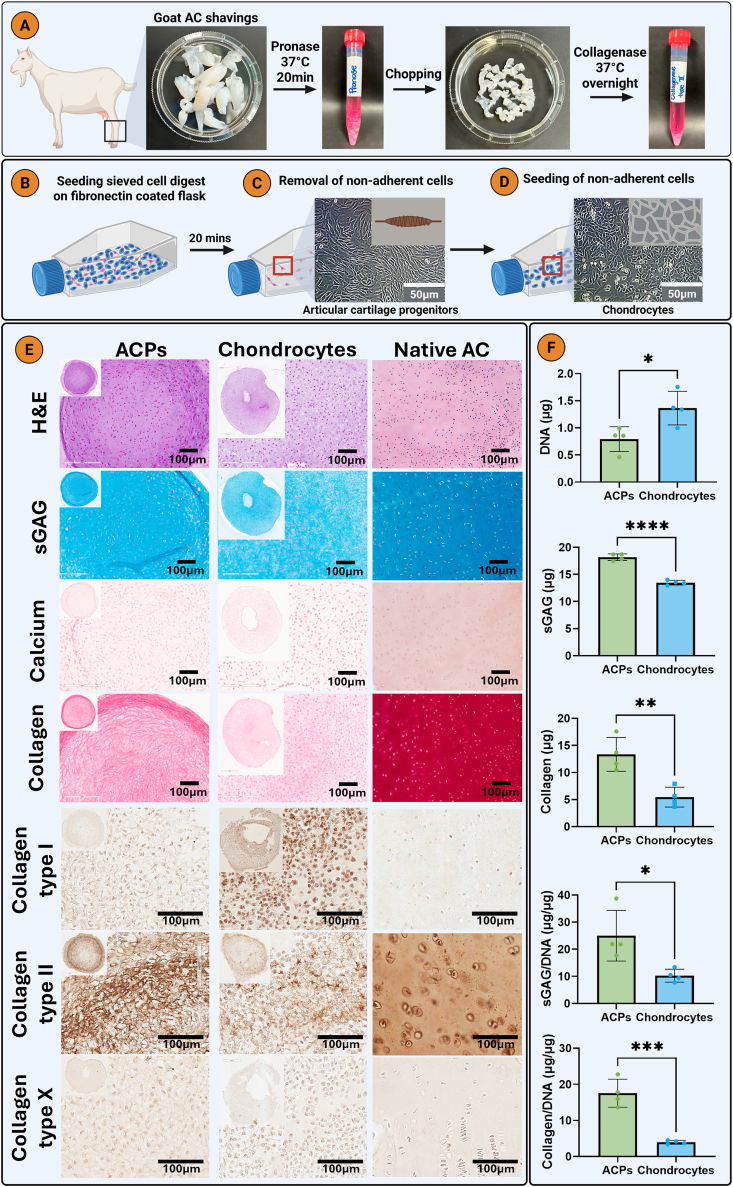
Isolation and characterization of articular cartilage progenitor cells (ACP) from full thickness AC shavings. A) Sequential enzymatic digestion of AC with pronase and collagenase. B) Isolation of progenitor cells through differential adhesion to fibronectin. C) Removal and D) seeding of non-adherent cells (chondrocytes). E) Chondrogenic differentiation of ACPs and chondrocytes in 3-week pellet culture. Histological staining with hematoxylin and eosin (H&E), picrosirius red (collagen), alcian blue (sGAG), and alizarin red (calcium). Immunohistochemistry for collagens type I, II and X. F) Biochemical quantification of DNA, sGAG, and collagen. A nonpaired student t-test was used for statistical analysis (n = 4). Significance levels: ∗p < 0.05, ∗∗p < 0.01, ∗∗∗p < 0.001, ∗∗∗∗p < 0.0001. Created with biorender.com.

**Fig. S2:**
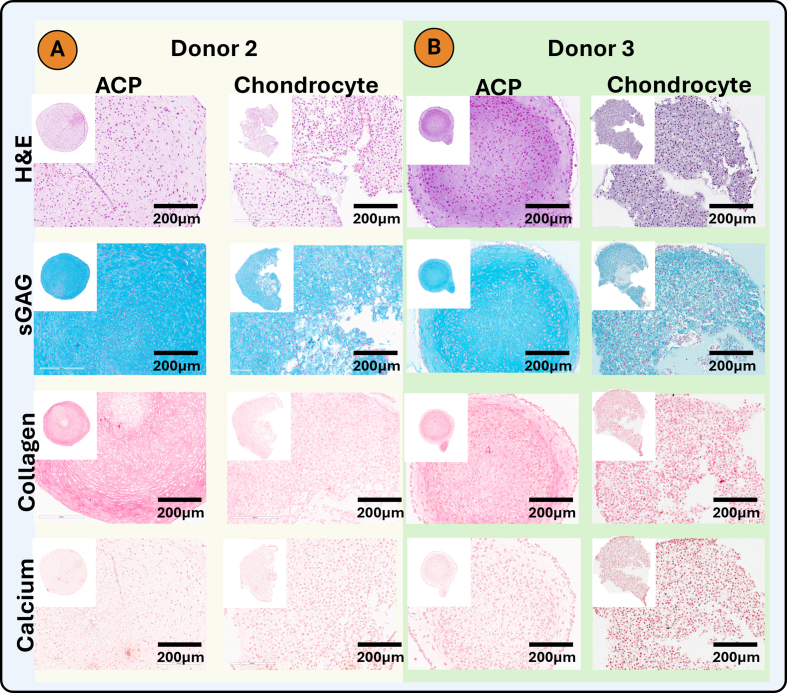
ACPs demonstrate superior chondrogenic differentiation in pellet culture when compared to donor matched chondrocytes following 3-week pellet culture. In both A) donor 1 and B) donor 2, ACPs stained more intensely for sGAG and collagen (n = 3).

In agreement to other studies [15], isolated ACPs displayed a spindle-like morphology (Fig. 1C), while chondrocytes took on a typical cobblestone-like shape (Fig. 1D) [46]. We first evaluated the chondrogenic differentiation of ACPs and chondrocytes in pellet culture. ACPs stained more intensely for sGAG and collagen (Fig. 1E) when compared to chondrocytes, which was comparable for different donors (Fig. S2). This was confirmed by biochemical assays, with ACPs secreting significantly more sGAG and collagen than chondrocytes (Fig. 1F). Additionally, the tissue produced by ACPs stained more intensely for type II collagen compared to chondrocytes (Fig. 1E). The pellets were also negative for type X collagen, indicating a hyaline cartilage phenotype. These results highlight the advantages of ACPs for cartilage TE applications. This agrees with previous studies demonstrating that ACPs secrete higher levels of sGAG compared to chondrocytes in pellet culture [15]. Similarly, when encapsulated in GelMA based hydrogels, ACPs have also been shown to produce a more hyaline cartilage matrix compared to chondrocytes [13,14].

**Fig. 2:**
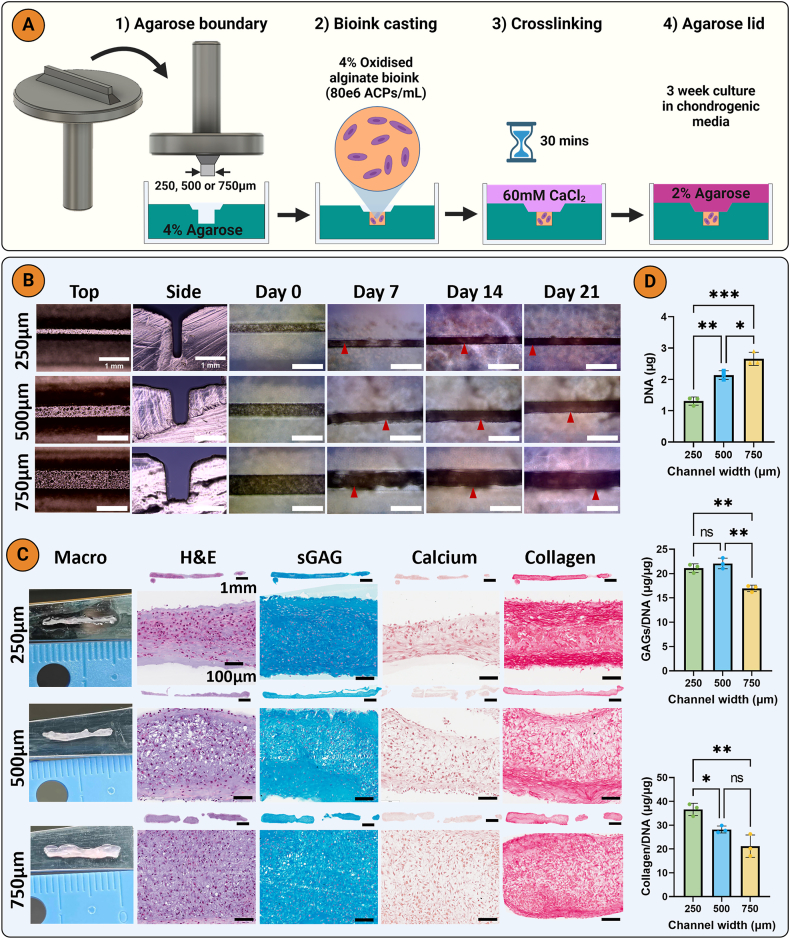
Thinner channel results in higher collagen/DNA A) Diagrammatic representation of the fabrication of agarose channels and bioink casting. B) Macroscopic images of agarose channels and casted bioinks. C) Histological staining with hematoxylin and eosin (H&E), picrosirius red (collagen), alcian blue (sGAG), and alizarin red (calcium). D) Biochemical quantification of DNA, sGAG, and collagen. An ordinary one-way analysis of variance (ANOVA) with Tukey's multiple comparisons was used for statistical analysis (n = 3). Significance levels: ns = not significant, ∗p < 0.05, ∗∗p < 0.01, ∗∗∗p < 0.001. Created with fusion 360 and biorender.com.

### 3.2 External physical boundaries influence the organization of collagen secreted by ACPs

**Fig. S3:**
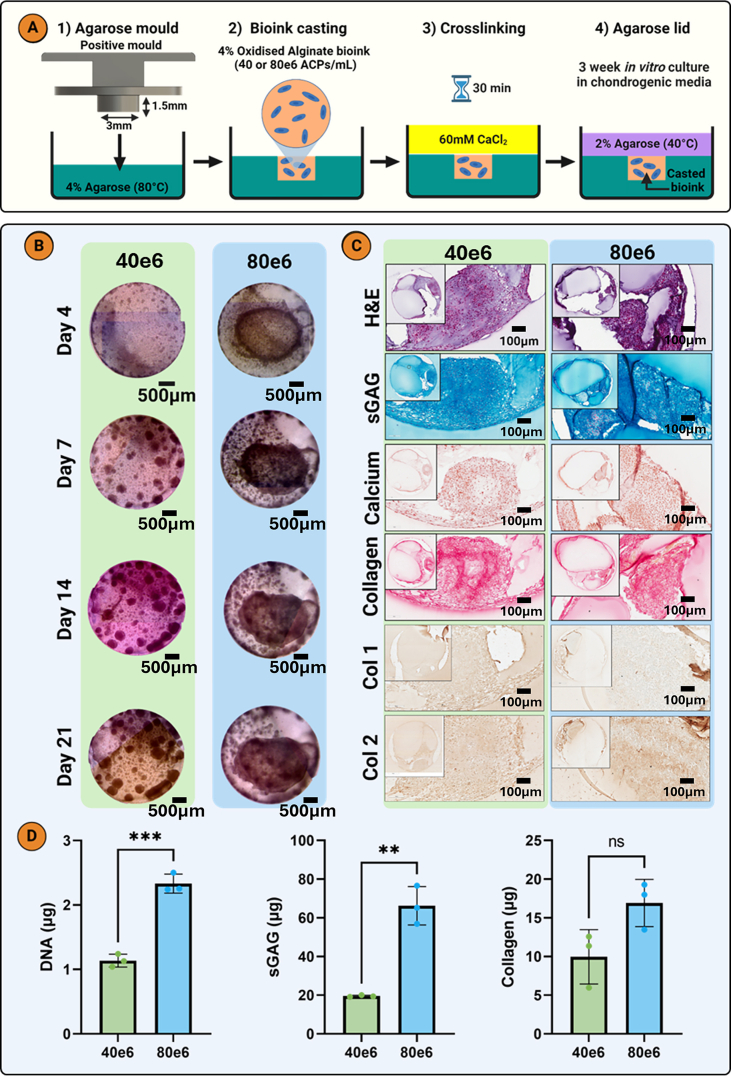
A) Diagrammatic representation of OA hydrogel casting of 40 or 80 million cells/mL in cylindrical agarose moulds (Ø = 3 mm). B) Brightfield images of casted hydrogel during 21-day *in vitro* culture. C) Histological staining with hematoxylin and eosin (H&E), picrosirius red (collagen), alcian blue (sGAG), and alizarin red (calcium). Immunohistochemistry for collagens type I and II. D) Biochemical quantification of DNA, sGAG, and collagen. A nonpaired student t-test was used for statistical analysis (n = 3). Significance levels: ns = not significant, ∗∗p < 0.01, ∗∗∗p < 0.001. Created with biorender.com.

We next sought to identify how ACPs would respond to different physical boundary conditions following encapsulation into OA hydrogels. Before subjecting ACPs to such biophysical cues, we first assessed how cell density (40 or 80x10^6^ ACPs/mL) would impact chondrogenesis in 4 % OA hydrogels (Fig. S3). OA hydrogels have previously been used as bioinks due to their tuneable degradation rate [44,47,48], enabling their use as a temporary scaffolding biomaterial that support cells as they begin secreting extracellular matrix [32,49]. The cell-laden OA hydrogels were cast in cylindrical agarose moulds (Fig. S3A) and cultured for 21 days. Following culture, only the 80x10^6^ density formed a cohesive tissue (Fig. S3B), with higher total sGAG and collagen content compared to the 40x10^6^ group (Fig. S3D). Building on this finding, the 80x10^6^ ACP/mL 4 % OA hydrogels were cast into agarose channels with a 250, 500, or 750 μm width to investigate the influence of varying boundary widths on collagen organization (Fig. 2A). The casted hydrogel contracted by day 7, but had begun to swell by day 14, filling the width of the channel with new cartilage tissue by day 21 (Fig. 2B, red arrows). After 21 days, the ACPs had formed handleable tissue filaments that could be manually removed from the channels using tweezers. Macroscopic images at day 21 showed the difference in width between the filaments (Fig. 2C). The 250 μm channel supported robust chondrogenesis, with more intense sGAG and collagen staining (Fig. 2C). Additionally, cells appeared to preferentially align parallel to the long axis of the channel in the 250 μm group. All groups stained negatively for calcium deposition (alizarin red staining), suggesting the ACPs were generating phenotypically stable AC (Fig. 2C). As expected, there was a significantly higher DNA content in the wider channels as they contained more cells (Fig. 2D). sGAG/DNA levels were significantly lower (p < 0.005) in the 750 μm channels than the other widths (Fig. 2D). Interestingly, the 250 μm channel supported significantly higher collagen/DNA when compared to the other channel widths (p < 0.005 and p < 0.05, respectively) (Fig. 2D).

**Fig. 3:**
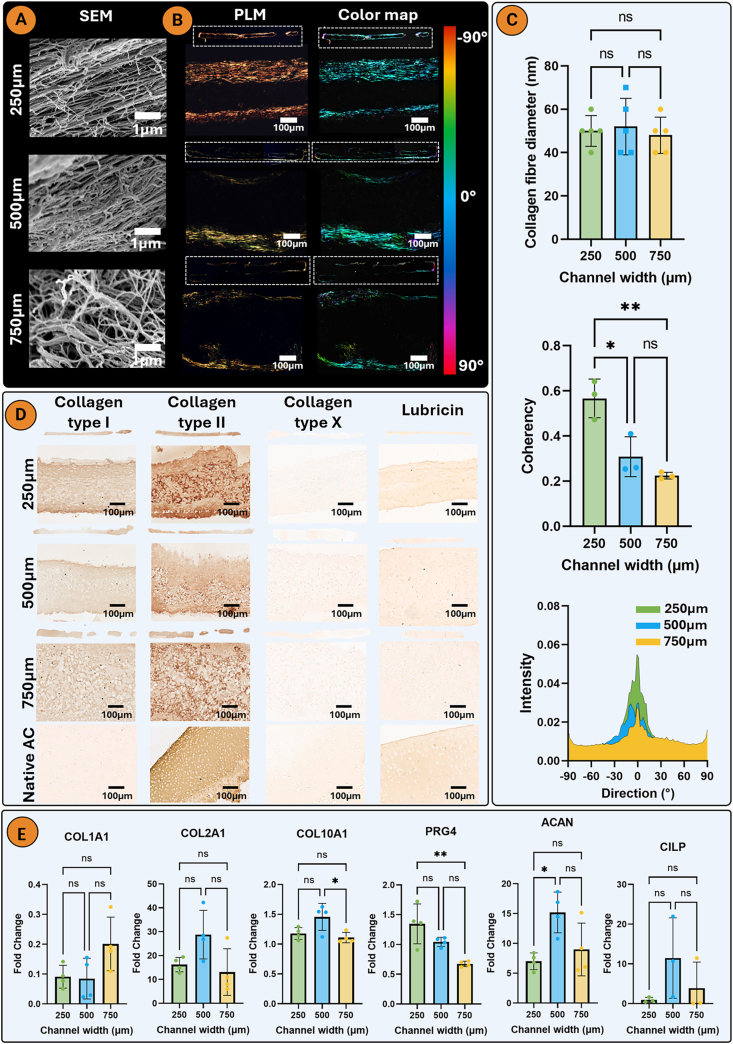
Thinner boundary spacing results in a higher neotissue collagen alignment A) Scanning electron microscopy (SEM) imaging of neotissue collagen of the engineered tissues in the different channel widths, B) Polarized light microscopy and color map imaging revealed an increased collagen alignment in the thinner 250 μm channel. C) Collagen fibre diameter (n = 5), coherency and intensity graphs (n = 3). D) Immunohistochemistry for collagens type I, II, and X and lubricin. E) Day 7 quantitative PCR for COL1A1, COL2A1, ACAN and CILP fold change relative to an ACP monolayer (n = 4). An ordinary one-way analysis of variance (ANOVA) with Tukey's multiple comparisons was used for statistical analysis. Significance levels: ns = not significant, ∗p < 0.05, ∗∗p < 0.01.

**Fig. S4:**
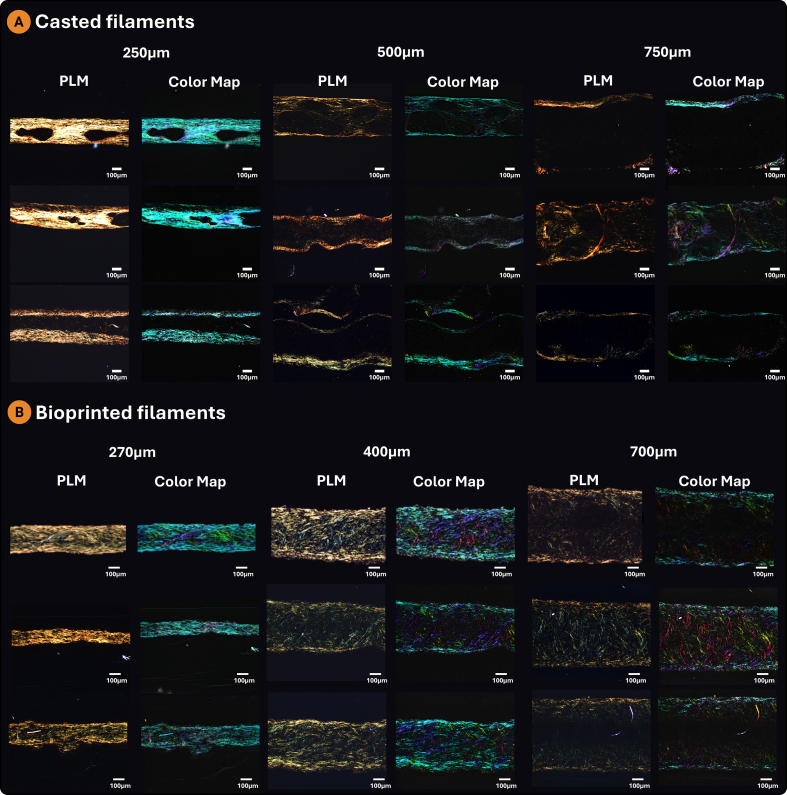
PLM analysis images of A) casted ACP hydrogel filaments and B) 3D bioprinted cell-only ACP filaments, shown at multiple section depths (n = 3).

**Fig. S5:**
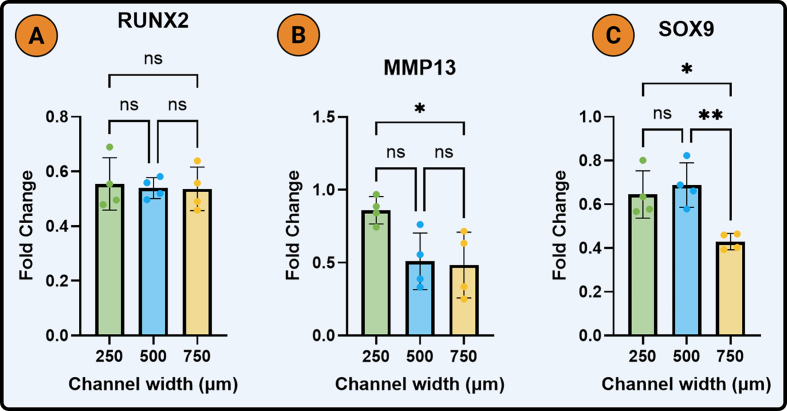
Day 7 quantitative PCR of casted ACP filaments in 250, 500 or 750 μm channel widths for A) RUNX2, B) MMP13, and C) SOX9. Fold change relative to an ACPs monolayer (n = 4). An ordinary one-way analysis of variance (ANOVA) with Tukey's multiple comparisons was used for statistical analysis. Significance levels: ns = not significant, ∗p < 0.05, ∗∗p < 0.01.

SEM images at day 21 indicated a higher collagen fibre alignment in the 250 μm channels, with a more random organization in the 750 μm channels (Fig. 3A). Similarly, PLM analysis revealed that the collagen fibrils adjacent to the agarose wall were aligned parallel to its long axis for all three channel widths (Fig. 3B). The greatest degree of collagen alignment was seen in the 250 μm group, where a more uniform and thicker region of alignment was observed compared to the wider channels. Additionally, the 250 μm channels promoted the highest fibre coherency, which is a coefficient used to evaluate the variance of fibre distribution (the closer the value is to 1, the lower the variance) (Fig. 3C; Fig. S4). There were no differences in collagen fibre diameter (Fig. 3C). Immunohistochemical analysis revealed a hyaline-like phenotype in all channel widths, with more intense staining for type II than type I collagen and negative staining for type X collagen (Fig. 3D). Quantitative PCR analysis showed elevated expression of ACAN and COL10A1 in the 500 μm channel width, while PRG4 and MMP13 expression were highest in the 250 μm width, consistent with the immunohistochemical findings (Fig. 3D and E; Fig. S5). No differences in RUNX2 gene expression were observed, while SOX9 expression was lowest in the 750 μm channel (Fig. S5).

**Fig. 4:**
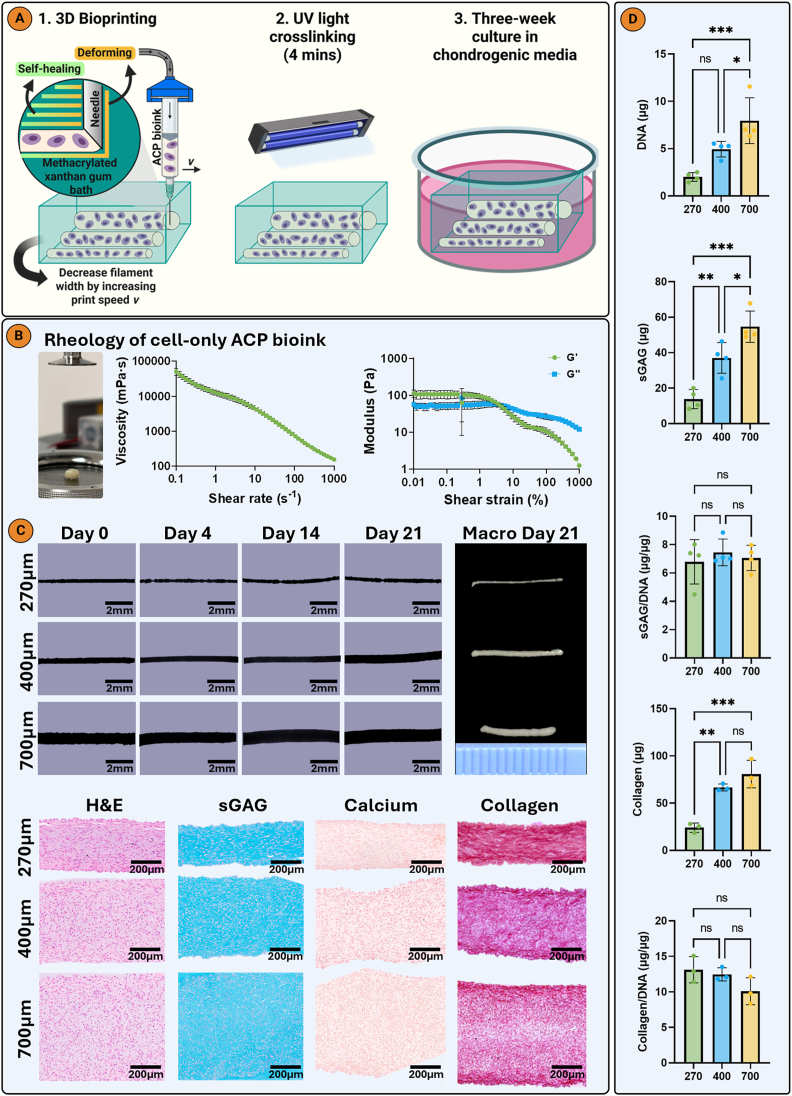
XGMA enables high resolution bioprinting and supports chondrogenesis of bioprinted ACPs. A) Diagrammatic representation of 3D bioprinting ACP filaments of varying widths in an XGMA support bath. B) Rheology of the ACP cell-only bioink indicating shear thinning behaviour. C) Brightfield images of bioprinted filaments during culture and macroscopic images at day 21. Histological staining with hematoxylin and eosin (H&E), picrosirius red (collagen), alcian blue (sGAG), and alizarin red (calcium). D) Biochemical quantification of DNA, sGAG, and collagen. An ordinary one-way analysis of variance (ANOVA) with Tukey's multiple comparisons was used for statistical analysis (n = 4). Significance levels: ns = not significant, ∗p < 0.05, ∗∗p < 0.01, ∗∗∗p < 0.001. Created with biorender.com.

The thinner agarose channels may have had a greater impact on collagen alignment due to the greater physical confinement forcing the cells and assembled collagen fibrils to align parallel to the long axis of the boundary. This has also been observed in 3D printed polycaprolactone (PCL) scaffolds that had elongated pores with different aspect ratios [50]. Following subcutaneous implantation in rats for 12 weeks, more organized tissue formed with scaffolds with higher aspect ratios. Similarly, we have previously shown that polymer scaffolds with larger aspect ratio pores supported the development of more organised tissue with significantly greater collagen fibre coherency [32]. Alternatively, the physical boundaries could be promoting alignment of the ACPs, which in turn might modulate collagen alignment. This was observed in tenocytes seeded on micropatterned grooves, which induced cell alignment in the direction of the long axis of the groove, followed by alignment of the secreted collagen [51]. This occurred despite no obvious difference in phenotype for cells seeded on the different groove widths. These findings demonstrate that external boundaries can be used to modulate neotissue collagen synthesis and alignment within ACP laden hydrogels.

### 3.3 A XGMA support bath functions as an external boundary to direct collagen organization in bioprinted cartilage

**Fig. S6:**
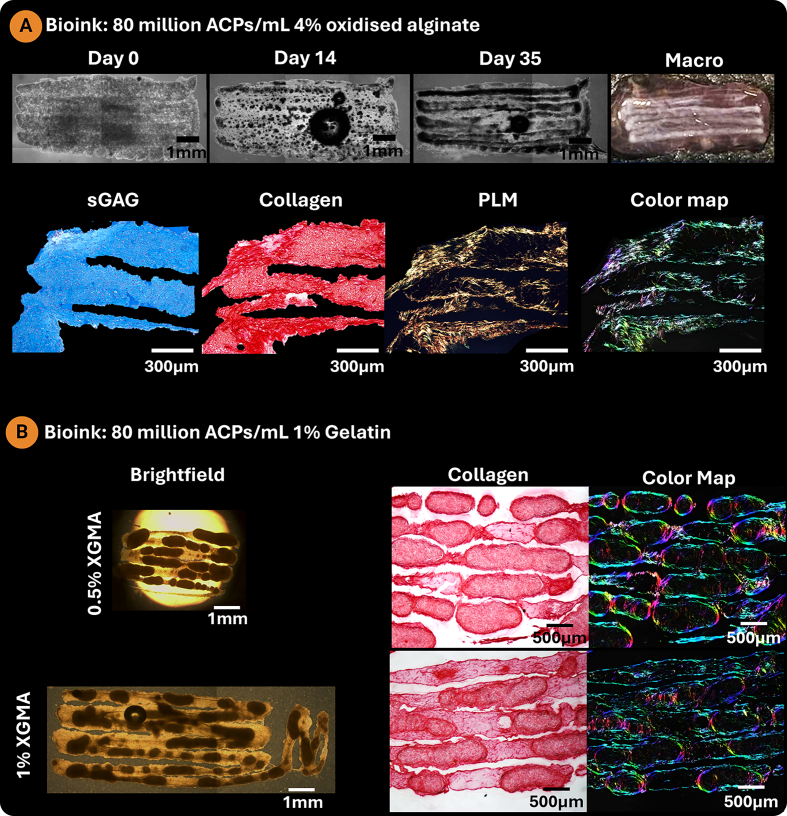
A) 3D bioprinting 80 million ACPs/mL 4 % (w/v) OA bioink. Brightfield images during *in vitro* culture in 1 % (w/v) XGMA. Histological staining with picrosirius red (collagen) and alcian blue (sGAG) and PLM imaging. B) 3D bioprinting 80 million ACPs/mL 1 % (w/v) gelatin bioink in 0.5 % or 1 % (w/v) XGMA. Brightfield images at day 28. Histological staining with picrosirius red (collagen) and PLM color map imaging.

We next sought to assess if the previous findings for ACPs within casted moulds of different widths could be translated into a 3D bioprinting framework. Filaments of different widths (270, 400 or 700 μm) were 3D bioprinted into a XGMA support bath (Fig. 4A) by adjusting the print speed [52]. Cells were printed without a supporting ink, sometimes termed ‘cell-only bioprinting’ [53], as poor collagen alignment was observed if cells were combined with a supporting OA ink (Fig. S6A), while the use of a 1 % (w/v) gelatin ink resulted in the formation of dispersed cellular aggregates rather than a cohesive tissue (Fig. S6B). The cell-only bioink possessed shear-thinning properties, making it ideal for extrusion bioprinting (Fig. 4B). The XGMA bath supported printed shape fidelity for up to 21 days of culture (Fig. 4C). All bioprinted filaments underwent robust chondrogenesis, with strong sGAG and collagen deposition and no evidence of calcification (Fig. 4C). Similar to the casted filaments, cells in the thinner diameter filaments appeared to preferentially align parallel to the long axis of the boundary. The larger diameter filaments had a higher DNA content due to their greater filament volume (Fig. 4D).

**Fig. 5:**
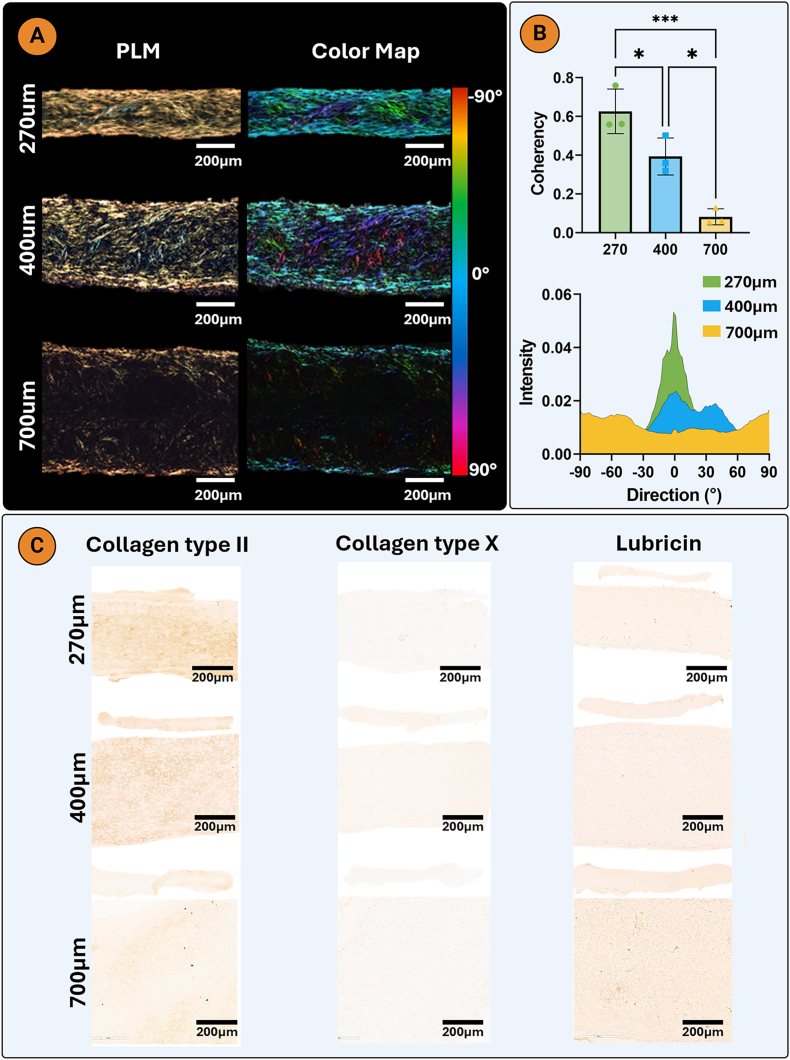
Thinner bioprinted filament supports greater neotissue collagen alignment A) Polarized light microscopy and color map imaging of bioprinted filaments of varying widths. B) Coherency and intensity graphs of collagen alignment. C) Immunohistochemistry for collagens type I, II, and X and lubricin. An ordinary one-way analysis of variance (ANOVA) with Tukey's multiple comparisons was used for statistical analysis (n = 3). Significance levels: ∗p < 0.05, ∗∗∗p < 0.001.

In agreement to the casted filaments, collagen fibre alignment and coherency was greatest in the thinner bioprinted filaments (Fig. 5A and B; Fig. S4). Physical shear stress during extrusion bioprinting is known to induce alignment of fibrillar additives in bioinks such as collagen or cellulose fibres [54]. Such aligned fibres can subsequently serve as guiding structures to direct cell alignment and neotissue growth. However, in this study no supporting hydrogel or bioink additives were added, thus it is hypothesized that the observed alignment is a direct result of the surrounding XGMA functioning as a physical boundary and providing a geometric constraint. All bioprinted filament widths were negative for type X collagen. Stronger collagen type II staining was observed in the thinner bioprinted filament, while a weak lubricin stain was observed in all filaments (Fig. 5C).

**Fig. 6:**
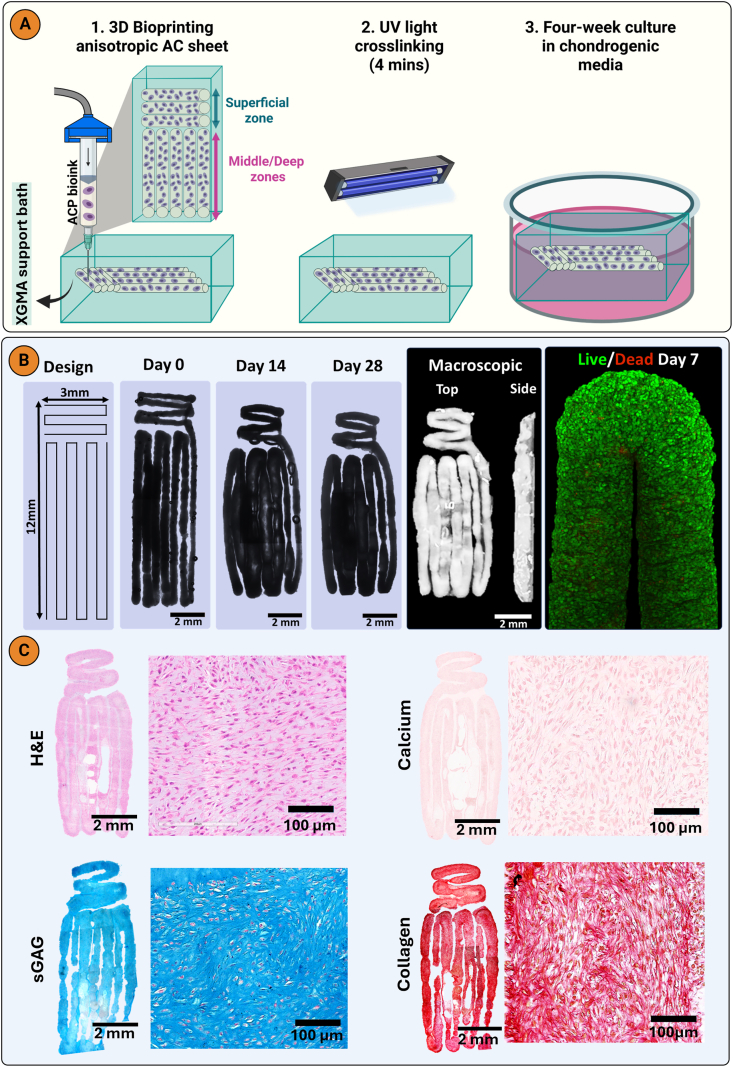
A) Diagrammatic representation of 3D bioprinting anisotropic AC sheet. B) Brightfield images of bioprinted sheet and Live/Dead imaging on Day 7 (n = 3). C) Histological staining with hematoxylin and eosin (H&E), picrosirius red (collagen), alcian blue (sGAG), and alizarin red (calcium). Created with biorender.com.

**Fig. 7:**
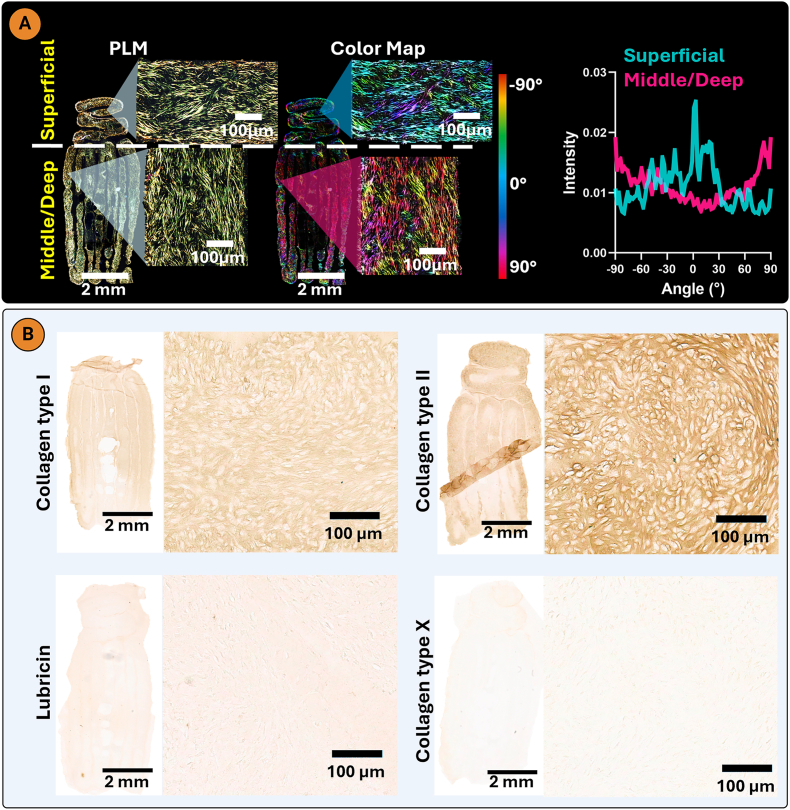
3D bioprinted AC sheet with an anisotropic collagen architecture. A) Polarized light microscopy and color map imaging of bioprinted AC sheet (n = 3). B) Immunohistochemistry for collagens type I, II, and X and lubricin.

**Fig. S7:**
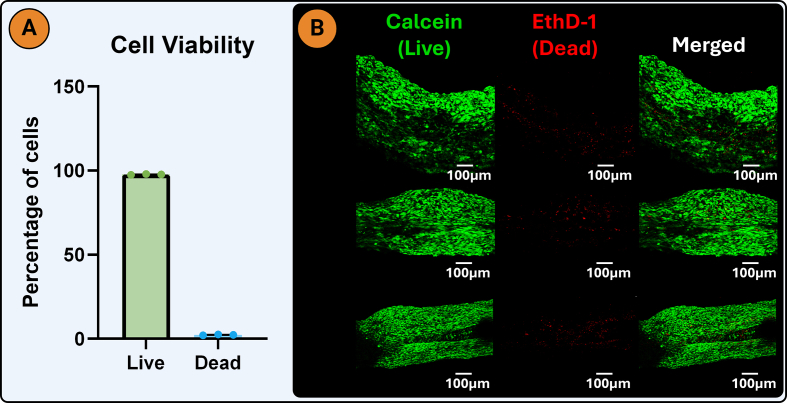
Cell viability analysis of ACP cell only bioprint at day 7. A) Percentage of live and dead cells. B) Confocal images of live and dead cells and merged channels (n = 3).

**Fig. S8:**
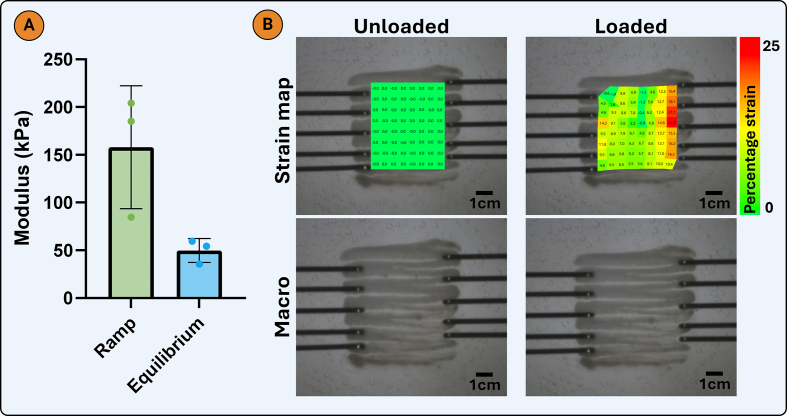
Uniaxial tensile mechanical testing of bioprinted ACP sheet. A) Ramp and equilibrium moduli and B) strain map analysis obtained during testing (n = 3).

Building on these findings, we next bioprinted a construct composed of horizontal and vertical filaments in an attempt to mimic aspects of the anisotropic collagen alignment observed in native AC (Fig. 6A). To enable the bioprinting of scaled-up grafts with minimal XGMA bath in the final construct, while still maintaining boundary induced alignment, 400 μm wide filaments were used. During bioprinting some contraction was observed along the long axis of each zone (Fig. 6B). After 7 days of culture, Live/Dead imaging confirmed that bioprinted ACPs had a high live cell viability of 97.73 % (Fig. 6B; Fig. S7). The tissue generated by the bioprinted ACPs was rich in sGAG and collagen, but negative for calcium deposits (Fig. 6C). PLM revealed an anisotropic collagen alignment in the bioprinted sheet, with a horizontal alignment within the horizontal filaments making up the superficial zone, and a vertical alignment in the vertical filaments of the middle/deep zones of the construct (Fig. 7A). In both zones, preferential collagen alignment was observed throughout the depth of the bioprinted filaments. A hyaline cartilage phenotype was maintained in the bioprinted sheet, which stained intensely for type II collagen and negative for type X collagen (Fig. 7B). This demonstrates that the XGMA support bath can function as a boundary between adjacent bioprinted filaments, enabling the engineering of larger, anisotropic tissues. The mechanical properties of the bioprinted cartilage sheet were evaluated under uniaxial tensile testing. The engineered cartilage exhibited a ramp modulus of 157.97 ± 52.45 kPa and an equilibrium modulus of 49.77 ± 10.25 kPa (Fig. S8).

Concerns about the potential toxicity and immunogenicity of biomaterials led to the development of scaffold-free approaches in 3D bioprinting. Early strategies used bioinks composed of preformed cellular aggregates, which were extruded to create desired geometries [55,56]. However, the resolution was limited by aggregate size and required an additional aggregation step. To overcome these limitations, cell-only bioprinting using highly concentrated cell suspensions has emerged, enabling the fabrication of complex geometries with micrometer-scale resolution [57]. In cartilage TE, MSC only bioprinting has shown promise, with constructs exhibiting positive sGAG staining after *in vitro* culture in chondrogenic media [53,58]. However, these studies did not assess collagen deposition and organization, which are key determinants of tissue functionality. Notably, 3D bioprinting in these contexts was used primarily to define the macroscopic shape of constructs, without addressing internal tissue architecture. In contrast, we now demonstrate that embedded ACP-only bioprinting can strategically introduce internal boundaries alongside extruded filaments, guiding the development of the resulting tissue to produce more biomimetic collagen architectures. These findings further show that a support bath can not only facilitate high-resolution printing of cell-only bioinks, but also direct extracellular matrix (ECM) alignment to enhance neotissue functionality.

### 3.4 3D bioprinting of AC grafts with an arcade-like collagen alignment

**Fig. 8:**
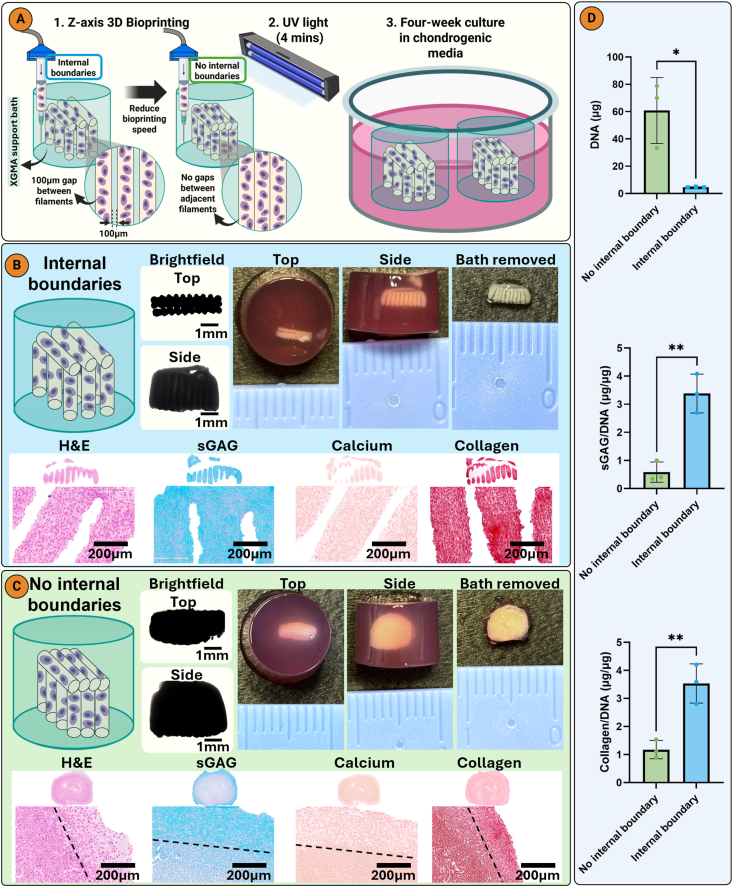
Internal boundaries enable robust chondrogenesis in bioprinted grafts. A) Diagrammatic representation of 3D bioprinting 2-layered AC sheet with or without internal boundaries. Brightfield and macroscopic images at day 21 of B) Internal boundaries and C) no internal boundaries constructs and histological staining with hematoxylin and eosin (H&E), picrosirius red (collagen), alcian blue (sGAG), and alizarin red (calcium). D) Biochemical quantification of DNA, sGAG, and collagen. A nonpaired student t-test was used for statistical analysis (n = 3). Significance levels: ∗p < 0.05, ∗∗∗p < 0.01. Created with biorender.com.

**Fig. S9:**
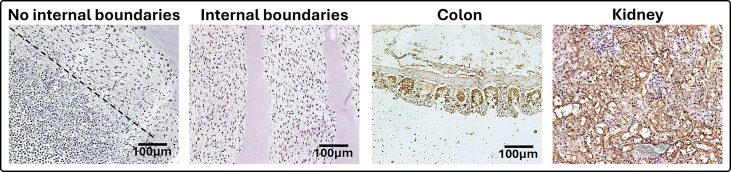
Immunohistochemistry of HIF-1α for ACP cell only bioprints with and without internal boundaries. Nuclei counterstained with hematoxylin. Colon and kidney tissue positive controls (n = 3).

To further scale up from the bioprinted sheet, we next employed Z-axis bioprinting for the middle/deep zone and XY-plane bioprinting for the superficial zone (Fig. 8A). This approach allowed us to create horizontal filaments overlaid on vertical filaments, either with or without internal XGMA boundaries, by adjusting the bioprinting speed. In the internal boundaries group, a 100 μm gap of XGMA was maintained between adjacent filaments, whereas in the no internal boundaries group, filaments were printed without any gaps (Fig. 8A). After 4 weeks of culture in chondrogenic media, constructs with internal boundaries showed uniform deposition of sGAG and collagen throughout the structure, indicating robust chondrogenesis (Fig. 8B). In contrast, sGAG and collagen was only observed at the periphery of constructs lacking internal boundaries, with limited matrix deposition and poor chondrogenesis in the centre (Fig. 8C). These findings highlight an additional advantage of incorporating internal boundaries, which is improved chondrogenesis in the core of the construct. This was further supported by biochemical analysis, which showed higher sGAG and collagen synthesis in constructs with internal boundaries. As expected, the total DNA content was higher in the no-boundary group, likely due to a greater number of printed cells (Fig. 8D). This increased cellularity may have contributed to nutrient competition and the observed reduction in chondrogenesis [59]. We did not observe any differences in HIF-1α staining between the groups (Fig. S9), suggesting that the addition of internal boundaries is not influencing the extent of hypoxia (or otherwise) developing in the constructs, although differential gradients in other key nutrients (e.g. glucose) or regulatory factors may develop. Computational models of oxygen and glucose diffusion in bioprinted constructs with and without internal channels have previously shown that the presence of the channels improved nutrient distribution and prevented core necrosis [60]. Our results are also in agreement with previous studies which found that deliberately introducing channels in engineered AC grafts enhanced tissue growth and matrix secretion [[61], [62], [63], [64]]. We hypothesise that a longer culture period for the internal boundaries group will facilitate tissue ingrowth into the ‘channels’ or spaces between the bioprinted filaments [64]. Future studies will directly test this hypothesis.

**Fig. 9:**
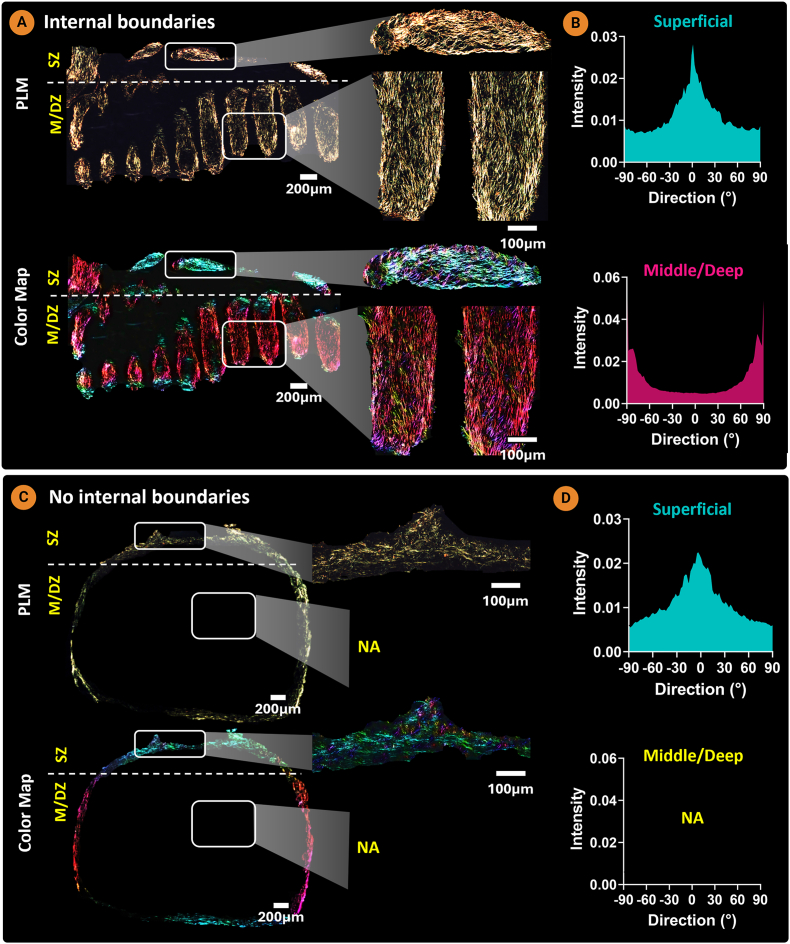
A) Internal boundaries enable biomimetic arcade-like collagen architecture in bioprinted constructs as revealed by PLM imaging. B) Intensity graph for the superficial and middle/deep zones. C) PLM imaging reveals Circumferential collagen alignment along the exterior of the no internal boundaries group. D) intensity graph for the superficial and middle/deep zones (n = 3).

Polarized light microscopy (PLM) revealed a biomimetic collagen alignment in the internal boundary group, with horizontal collagen fibres in the superficial zone and vertical fibres in the middle/deep zone (Fig. 9A). Conversely, circumferential collagen alignment localized to the external edges was observed in the no-boundary construct (Fig. 9C). These results suggest that internal boundaries not only facilitate chondrogenesis in cell-dense bioprints, but also guide collagen alignment along the longitudinal axis of the boundaries. The concept of using internal boundaries to guide collagen organization in engineered grafts has also been applied in other biofabrication strategies. For example, the Filament light (Flight) approach employs aligned microfilaments and microchannels created in a photosensitive hyaluronic acid-based bioresin, serving as physical boundaries that guide tissue architecture [65]. A comparison group without internal microchannels showed reduced sGAG and collagen synthesis and disrupted alignment, in agreement with our findings. Similarly, decellularized extracellular matrix scaffolds with anisotropic pore structures have been shown to direct collagen organization into arcade-like patterns [24].

Collectively these results point to the importance of biophysical cues in the engineering of structurally organised AC. Key limitations of this work include the presence of the internal boundaries of XGMA support bath in the final graft and the absence of mechanical characterization of the bioprinted constructs. Future work will focus on modifying the support bath to degrade it at a predefined stage, allowing filament fusion and tissue ingrowth while preserving collagen alignment during the critical early phases of tissue formation. Additionally, further comprehensive mechanical testing will be essential to validate the functional performance of the bioprinted grafts. It would be interesting to also investigate the mechanistic pathways behind the observed collagen alignment. However, it is important to note that while we were able to engineer anisotropic cartilage using this approach the resulting grafts do not fully recapitulate the complete collagen network complexity of AC nor zonal cellular morphology. Future studies exploring the addition of biochemical cues within the bath could be used to potentially address this. Overall, our findings highlight that confinement within a support bath can reliably direct neotissue collagen organization, with thinner filaments further enhancing alignment. This approach offers a promising path toward 3D bioprinting of structurally organized, scaffold-free, and truly functional AC grafts.

## 4 Conclusions

This study demonstrates that physical boundaries, such as agarose or XGMA, can effectively direct the alignment of collagen secreted by ACPs. It was evident that the thinner casted and bioprinted filaments had a higher degree of collagen alignment compared to the thicker filaments. We successfully scaled this approach, from bioprinting single filaments to a multi-layered graft, where the XGMA support bath served as a boundary between adjacent filaments. This strategy enabled the fabrication of a graft with an arcade-like collagen architecture: horizontally aligned fibres in the superficial layer overlaying vertically aligned fibres in the deeper zone, thus recapitulating key features of native AC.

## CRediT authorship contribution statement

**Aliaa S. Karam:** Writing – review & editing, Writing – original draft, Visualization, Validation, Supervision, Methodology, Investigation, Formal analysis, Data curation, Conceptualization. **Gabriela S. Kronemberger:** Writing – review & editing, Validation, Supervision, Methodology, Investigation, Conceptualization. **Kaoutar Chattahy:** Writing – review & editing, Methodology, Investigation. **Daniel J. Kelly:** Writing – review & editing, Validation, Supervision, Project administration, Methodology, Funding acquisition, Conceptualization.

## Ethics approval and consent to participate

The caprine articular cartilage shavings for articular cartilage progenitor cell isolation were obtained from animals euthanized as part of a separate, ethics-approved project by the Irish Health Products Regulatory Authority (approval number AE18982).

## Funding

This work was funded by the European Research Council (ERC, 4D-BOUNDARIES #101019344).

## Declaration of competing interest

The authors declare no conflict of interest.
