# Supplementary material for: Cell-only bioprinting of articular cartilage progenitor cells within a physically constraining support bath to engineer structurally organized grafts
Source: Bioact Mater. 2025 Dec 29;59:251–65. doi: 10.1016/j.bioactmat.2025.12.013 (PMC12803855; doi:10.1016/j.bioactmat.2025.12.013)
Supplement: Multimedia component 1 [file mmc1.docx]

**Article title**

Cell only bioprinting in a photocrosslinkable support bath - A strategy to 3D bioprint structurally organised anisotropic tissues

**Authors**

A. S. Karam^1 ,2, 3^, G. S. Kronemberger^1 ,2, 3^, K. Chattahy^1 ,2, 3^, G. Gonnella^1 ,2, 3^, D. J. Kelly^1 ,2, 3, 4,^ *

**Affiliations**

^1^ Trinity Centre for Biomedical Engineering, Trinity Biomedical Sciences Institute, Trinity College Dublin, Dublin D02 PN40, Ireland.

^2^ Department of Mechanical, Manufacturing and Biomedical Engineering, School of Engineering, Trinity College Dublin, Dublin, Ireland.

^3^ Advanced Materials and Bioengineering Research Centre (AMBER), Royal College of Surgeons in Ireland and Trinity College Dublin, Dublin, Ireland.

^4^ Department of Anatomy and Regenerative Medicine, Royal College of Surgeons in Ireland, Dublin, Ireland.

*Corresponding Author

**Corresponding author’s email address and Twitter handle**

Email address: kellyd9@tcd.ie

X handle: @dannykelly1978

**Keywords**

Cell-only bioink; Boundary conditions; Methacrylated xanthan gum; Collagen alignment

**Related research article**

A. S. Karam, G. S. Kronemberger, K. Chattahy, D. J. Kelly, Cell-Only Bioprinting of Articular Cartilage Progenitor Cells within a Physically Constraining Support Bath to Engineer Structurally Organized Grafts, Bioactive Materials (2025).

**Abstract**

Even though the articular cartilage (AC) is only around 2mm in thickness, it has a highly structured extracellular matrix that is integral to its function. Specifically, the unique arcade-like collagen architecture of AC is crucial for its load-bearing properties allowing it to resist applied stresses and facilitate locomotion of the body. Three-dimensional (3D) bioprinting has recently emerged as a promising biofabrication technique capable of producing tissue constructs that closely mimic the native structural and cellular organization of AC. Unlike scaffold-based methods, cell-only bioprinting leverages the intrinsic ability of progenitor cells to self-assemble and remodel their environment, particularly when provided with well-defined physical and mechanical cues. Herein, we describe a technique of cell-only extrusion bioprinting of articular cartilage progenitor cells (ACPs) within a physically constraining methacrylated xanthan gum (XGMA) support bath, which directs cell alignment and promotes neotissue collagen organization along the long axis of the boundary. This approach will pave the way to enable the 3D bioprinting of structurally organised and hence functional tissues.

- Extrusion 3D bioprinting of a cell-only bioink in XGMA enables high resolution bioprinting while also functioning as a physical boundary that directs collagen alignment.

**Graphical abstract**

**
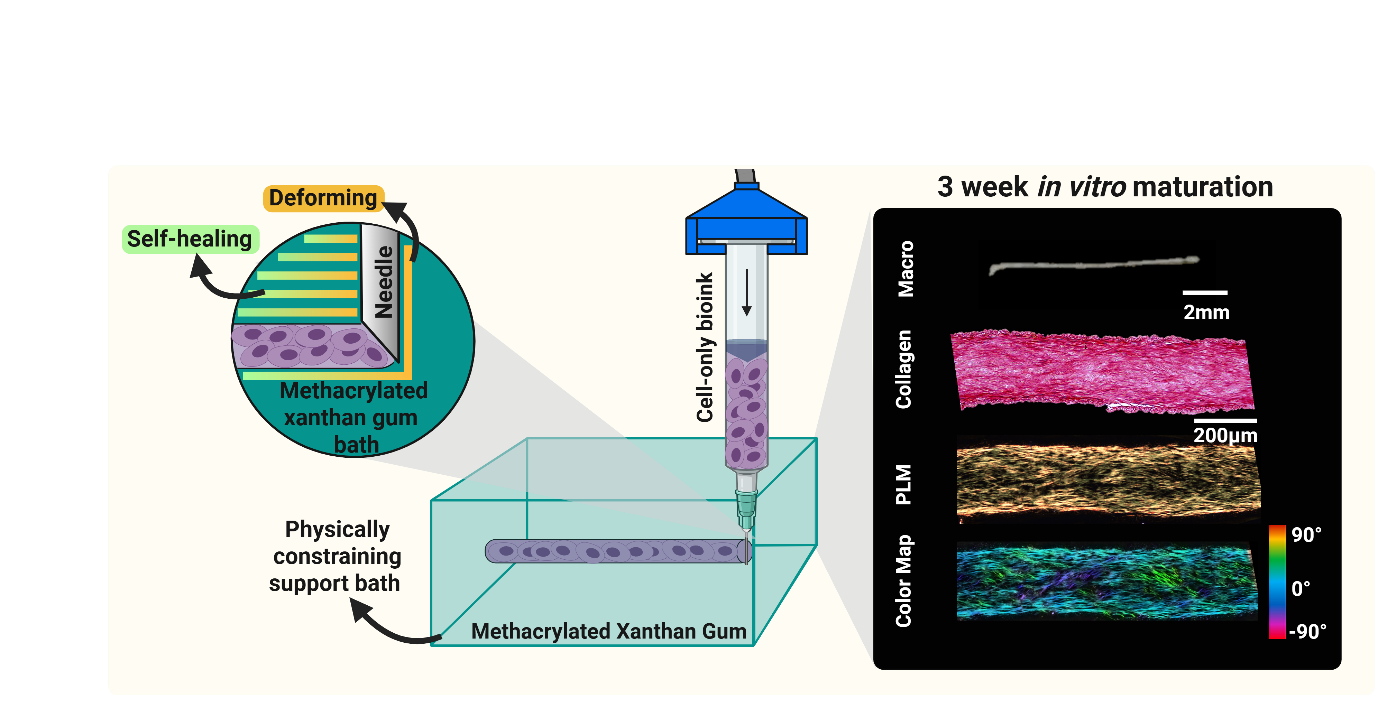
**

**Specifications table**

| **Subject area** | Materials Science |
| --- | --- |
| **More specific subject area** | Tissue engineering and 3D bioprinting |
| **Name of your method** | Cell only 3D extrusion bioprinting of articular cartilage progenitor cells in a physically constraining support bath to direct neotissue growth and organization |
| **Name and reference of original method** | O. Jeon, Y.B. Lee, H. Jeong, S.J. Lee, D. Wells, E. Alsberg, Individual cell-only bioink and photocurable supporting medium for 3D printing and generation of engineered tissues with complex geometries, Mater. Horiz. 6 (2019) 1625–1631. https://doi.org/10.1039/c9mh00375d  S. Patrício, L. Sousa, T. Correia, et al., Freeform 3D printing using a continuous viscoelastic supporting matrix, Biofabrication 12 (2020) 035017. https://doi.org/10.1088/1758-5090/ab8bc3 |
| **Resource availability** |  |

**Background**

Tissues throughout the human body have a highly structured extracellular matrix (ECM) that is fundamental to their function. For example, the unique arcade-like collagen architecture seen in the articular cartilage (AC) is crucial for its load-bearing properties, allowing it to resist applied stresses and facilitate locomotion [1, 2]. While multilayered scaffolds and hydrogels have been developed to mimic certain aspects of the zonal composition of AC [3-12], they typically fail to produce engineered tissues with an arcade-like collagen network. Computational modelling has shown that achieving this collagen organisation is the most important parameter in determining the functional success of an engineered AC graft [13]. This motivates the need for innovative tissue engineering strategies that can direct collagen alignment and, hence, generate functional AC grafts. Emerging 3D bioprinting techniques could potentially be used to address limitations of classical tissue engineering strategies by providing spatially and temporally defined biochemical and biophysical cues to cells as they self-organise into a structurally organised tissue. These cues or boundaries would serve as guiding structures for neotissue growth and cell alignment. Herein, we describe a technique of cell-only extrusion bioprinting of articular cartilage progenitor cells (ACPs) within a physically constraining methacrylated xanthan gum (XGMA) support bath, which directs cell alignment and promotes neotissue collagen organization along the long axis of the boundary [14].

**Method details**

***Isolation of ACPs***

Cell culture flasks were coated with 10µg/mL human fibronectin (Brennan & Co) in 0.1M Dulbecco’s phosphate buffer (PBS) with 1mM MgCl and 1mM CaCl_2_ overnight at 4°C (all Sigma, Ireland) [15]. On the following day the fibronectin solution is removed from the flasks, and they are left to air dry for 1-3 hours in the cell culture hood. The coated flasks can be stored at 4°C until use. The remaining fibronectin solution can be stored for up to one week at 4°C to coat other flasks. ACPs were isolated from full thickness AC shavings of skeletally mature female goats under sterile conditions (Fig. 1).


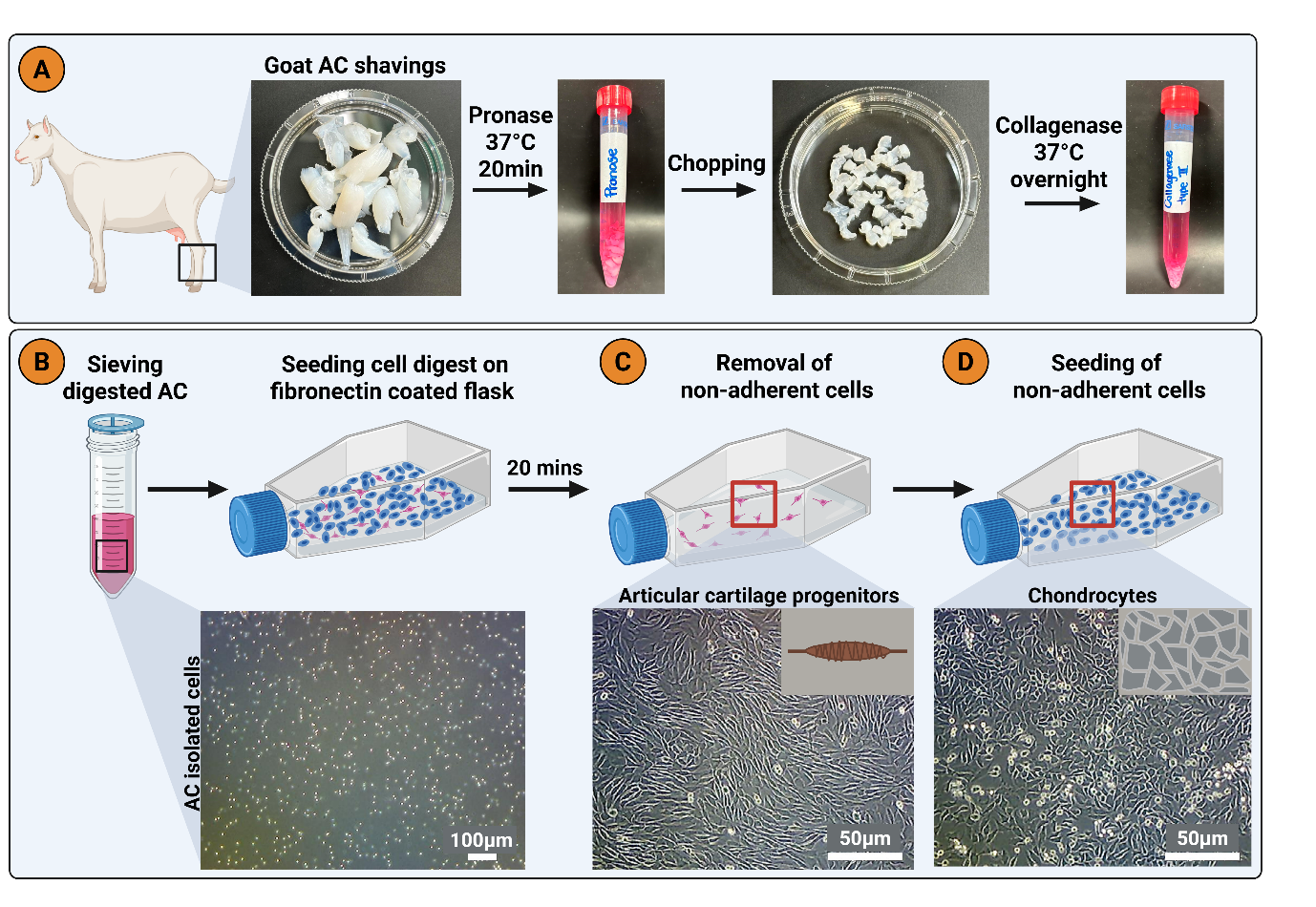


***Figure 1*** Isolation of articular cartilage progenitor cells (ACP) from full thickness AC shavings. A) Sequential enzymatic digestion of AC with pronase and collagenase. B) Sieving of tissue digest to remove any undigested tissue clumps followed by isolation of progenitor cells through differential adhesion to fibronectin. C) Removal and D) seeding of non-adherent cells (chondrocytes).

To isolate ACPs the AC was rinsed twice with PBS containing 100U/mL penicillin, 100μg/mL streptomycin and 2.5μg/mL amphotericin B and then digested in pronase (70U/mL, 2.7mL/g of AC, ThermoFischer Scientific) for 30 minutes at 37°C. Using a scalpel, the AC shavings were chopped into approximately 1-2mm^3^ pieces. The minced AC was then digested in collagenase type II (300U/mL, Gibco, Ireland, 8mL/g of AC) overnight at 37°C under continuous rotation (40RPM). All enzymes were prepared in serum free Dulbecco's Modified Eagle Medium/Nutrient Mixture F-12 (DMEM/F-12, ThermoFischer Scientific) supplemented with 100U/mL penicillin, 100μg/mL streptomycin, and 2.5μg/mL amphotericin B (all Gibco, Biosciences, Dublin, Ireland). On the following day, the digested AC was filtered through a 70µm sieve to remove any undigested debris. The sieved digest was centrifuged at 500RPM for 5 minutes. The supernatant was disposed, and the cell pellet was resuspended in serum free DMEM/F-12 supplemented with 100U/mL penicillin, 100μg/mL streptomycin, and 2.5μg/mL amphotericin B. The cells were then counted and seeded into the fibronectin coated flasks. After 20 minutes, non-adherent cells (chondrocytes) were removed, counted, and replated into non-fibronectin coated flasks and cultured in expansion media (XPAN) composed of high-glucose DMEM (Bioscience, Ireland) supplemented with 10% (v/v) fetal bovine serum (FBS, GIBCO, Biosciences, Ireland), 100U/mL penicillin (Biosciences, Ireland), 100μg/mL streptomycin (Biosciences, Ireland), and 2.5μg/mL amphotericin B (Sigma-Aldrich, Ireland). Percentage adherence was calculated (Table 1).

***Table 1*** Percentage adherence of cells from full thickness goat articular cartilage shavings

| **Donor** | **Articular cartilage shavings (g)** | **Total number of cells** | **Adherence (%)** |
| --- | --- | --- | --- |
| **Donor 1** | 1.44 | 4,170,000 | 13.7 |
| **Donor 2** | 1.5 | 3,375,000 | 30 |
| **Donor 3** | 2.7 | 5,300,000 | 35 |

The adherent cells (ACPs) were cultured in XPAN-12 composed of DMEM/F-12 (ThermoFischer Scientific) supplemented with 10% (v/v) FBS (GIBCO, Biosciences, Ireland), 100U/mL penicillin (Biosciences, Ireland), 5ng/mL of FGF-2 (PeproTech), 100μg/mL streptomycin (Biosciences, Ireland), and 2.5μg/mL amphotericin B (Sigma-Aldrich, Ireland). On day 2, ACPs were switched to A_XPAN composed of XPAN-12 supplemented with 0.5µg/mL of L-ascorbic acid 2-phosphate (Sigma-Aldrich, Ireland) and 1ng/mL of TGF-β1 (PeproTech, USA). Media changes for the chondrocytes and ACPs were performed every 2-3 days. After colonies were 80% confluent, the chondrocytes and ACPs were harvested using trypsin (Sigma-Aldrich, Ireland) and TrypLE (Biosciences, Ireland), respectively and then expanded until passage 4 at 5% O_2_ stimulation. ACPs display a spindle-like morphology while chondrocytes have a characteristic cobblestone-like shape (Fig. 1C and D). At each passage population doublings of ACPs and chondrocytes were calculated (Table 2).

***Table 2*** Population doublings of ACPs and chondrocytes from passage 0 to passage 4 of donor 1

| **Cell type** | **Passage** |  | **Population doubling** |
| --- | --- | --- | --- |
| **ACPs** | P0-P1  P1-P2  P2-P3  P3-P4 |  | 3.73  2.37  2.83  3.7 |
| **Chondrocytes** | P0-P1  P1-P2  P2-P3  P3-P4 |  | 2.45  3.13  2.11  2.5 |

***Preparation and storage of XGMA***

To prepare the methacrylated xanthan gum (XGMA) bioprinting support bath [16, 17] 0.5g of xanthan gum (XG, Sigma, Ireland) was dissolved in 100mL distilled water while stirring overnight at room temperature. Afterwards, 4mL of glycidyl methacrylate (Sigma, Ireland) was added to the XG at 60°C overnight while stirring in the dark. The solution was dialysed (MWCO 6-8kDa) for one week at room temperature in the dark while changing the water 3 times per day. Subsequently, the solution was freeze dried and stored at -20°C. For sterile bioprinting the freeze dried XGMA was sterilized with ethylene oxide gas and stored at -20°C until use. For bioprinting XGMA (1% (w/v)) was dissolved in phenol free DMEM supplemented with 100U/mL penicillin, 100μg/mL streptomycin, and 2.5μg/mL amphotericin B. Lithium phenyl-2,4,6-trimethylbenzoylphosphinate (LAP) (Sigma, Ireland) was added as a photoinitiator at a 1:10 ratio relative to the media volume and wrapped in foil to prevent crosslinking. The XGMA is left to dissolve for 2 days on a rotator at 40RPM in room temperature protected from light. Once dissolved the XGMA is centrifuged at 650G for 5 minutes to remove any bubbles and then stored at 4°C for up to 10 days. To bioprint multi-layered constructs, whilst still having space for cell culture media, cloning rings (Sigma, Ireland) can be used to house the bath during bioprinting. A viscous pipette is used to pipette XGMA into the cloning rings to prevent bubble formation. After bioprinting and XGMA UV crosslinking, wells are flushed with media and the cloning rings can be easily removed with tweezers (Fig. 2A). Alternatively, in house made PDMS rings can be used which are autoclavable and reusable (Fig. 2B).


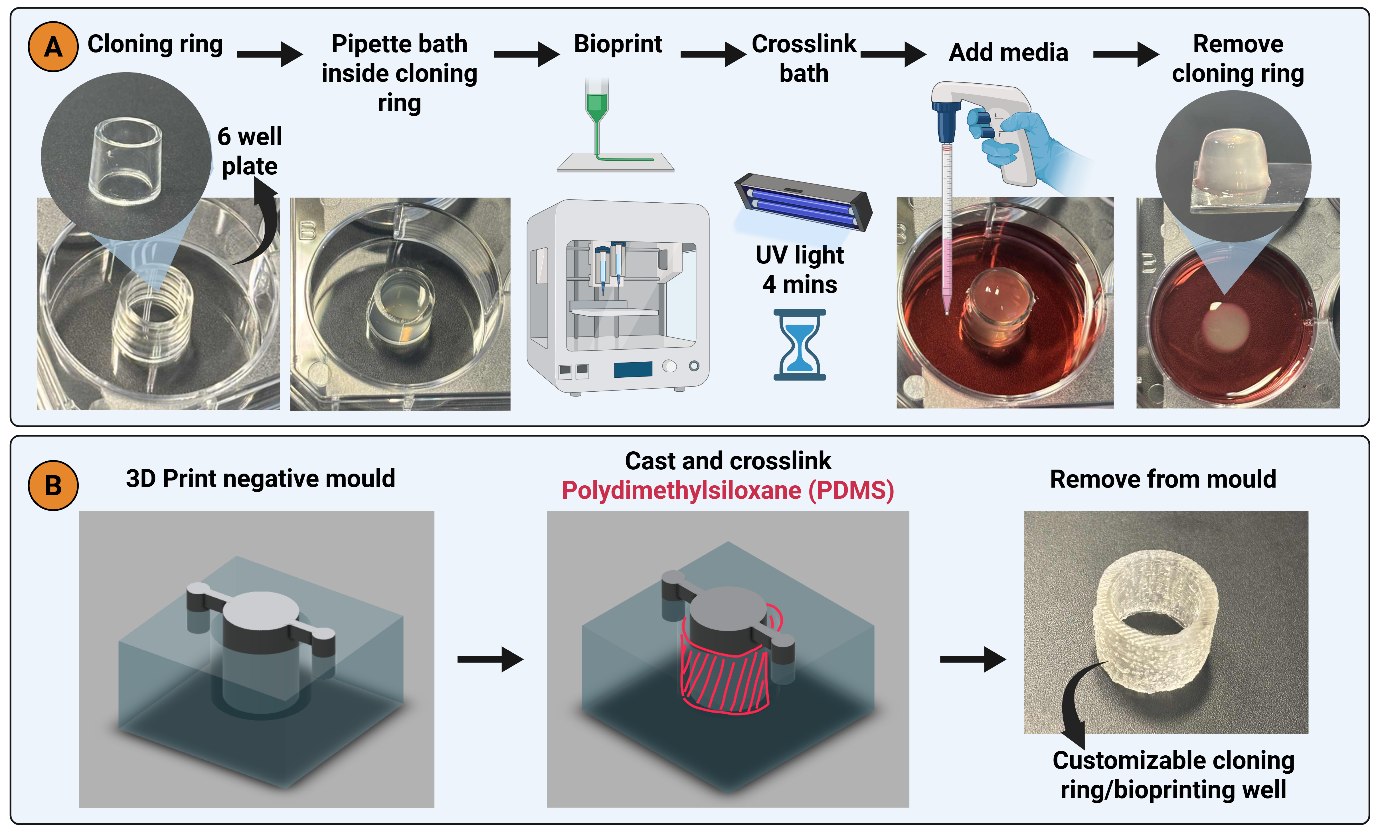


***Figure 2*** A) The use of cloning rings to create a smaller well within a 6-well plate for embedded 3D bioprinting with XGMA. Post crosslinking the cloning rings can be easily removed leaving space for media. B) One can fabricate any cloning ring shape needed with PDMS.

***Cell-only extrusion bioprinting***

For cell-only bioprinting of ACPs 40 T175 flasks will yield 700µL of bioink equivalent to around 350 million ACPs. To reduce space needed for expansion five layered flasks (scientific laboratory supplies, Ireland) are used. Once ACPs are detached with TrypLE they are combined in one 50mL tube and centrifuged at 650G for 5 minutes. From now on the tube containing the cell pellet is kept on ice. Using a stripette pipette bubbles remaining on the top of the supernatant are carefully aspirated first then the supernatant is aspirated carefully without disrupting the cell pellet. Any remaining supernatant can also be carefully aspirated with a P200 pipette. A viscous liquid pipette is used to aspirate the cell pellet and load it into the bioprinting syringe. This allows the transfer of the cell pellet without generating bubbles. For the 3D bioprinting a syringe pump printhead (CELLINK, Sweden) was used on the CELLINK BIO X6 (CELLINK, Sweden). A 3mL syringe barrel (BD Plastipak scientific laboratory supplies, Ireland) with a 2.5mL reduced dead space syringe plunger (Unifix UKMEDI, UK) were used for 3D bioprinting with a 25-gauge needle. Once the syringe is loaded with the cells it is kept in the fridge at 4°C for 20 to 30 minutes before bioprinting to prevent cell clumping.

**Method validation**

XGMA bioprinting support bath was used as a physical confinement to direct neotissue collagen growth of a cell-only ACP bioink where it was demonstrated that the greater physical confinement enhances cell and collagen alignment [14]. Hence, cell-only bioprinting of a scaled up zonally organized anisotropic cartilage graft was achieved [14].

**CRediT author statement**

Conceptualization: A.K, D.K.; Formal analysis: A.K; Funding acquisition: D.K; Investigation: A.K, G.K, K.C; Methodology: A.K, G.K, K.C, G.G, D.K.; Project administration: D.K; Supervision: A.K, G.K, D.K; Validation: A.K, G.K, D. K; Visualization: A.K; Writing - original draft: A.K, D.K.; Writing - review & editing: A.K, G.K, K.C, G.G, D. K.

**Acknowledgments**

Figures 1 and 2 were created with www.BioRender.com

**References**

[1] J. Owen, J. Wayne, Influence of a superficial tangential zone over repairing cartilage defects: implications for tissue engineering, Biomech. Model. Mechanobiol. 5 (2006) 102–110. https://doi.org/10.1007/s10237-006-0022-5

[2] R. Shirazi, A. Shirazi-Adl, Deep vertical collagen fibrils play a significant role in mechanics of articular cartilage, J. Orthop. Res. 26 (2008) 608–615. https://doi.org/10.1002/jor.20537

[3] J.A. Steele, S.D. McCullen, A. Callanan, H. Autefage, M.A. Accardi, D. Dini, M.M. Stevens, Combinatorial scaffold morphologies for zonal articular cartilage engineering, Acta Biomater. 10 (2014) 2065–2075. https://doi.org/10.1016/j.actbio.2013.12.030

[4] J.A. Steele, A.C. Moore, J.P. St-Pierre, S.D. McCullen, A.J. Gormley, C.C. Horgan, C.R. Black, C. Meinert, T. Klein, S. Saifzadeh, R. Steck, J. Ren, M.A. Woodruff, M.M. Stevens, In vitro and in vivo investigation of a zonal microstructured scaffold for osteochondral defect repair, Biomaterials 286 (2022) 121548. https://doi.org/10.1016/j.biomaterials.2022.121548

[5] M. Kim, M.J. Farrell, D.R. Steinberg, J.A. Burdick, R.L. Mauck, Enhanced nutrient transport improves the depth-dependent properties of tri-layered engineered cartilage constructs with zonal co-culture of chondrocytes and MSCs, Acta Biomater. 58 (2017) 1–11. https://doi.org/10.1016/j.actbio.2017.06.025

[6] M. Castilho, V. Mouser, M. Chen, J. Malda, K. Ito, Bi-layered micro-fibre reinforced hydrogels for articular cartilage regeneration, Acta Biomater. 95 (2019) 297–306. https://doi.org/10.1016/j.actbio.2019.06.030

[7] L. Fu, Z. Yang, C. Gao, H. Li, Z. Yuan, F. Wang, X. Sui, S. Liu, Q. Guo, Advances and prospects in biomimetic multilayered scaffolds for articular cartilage regeneration, Regen. Biomater. 7 (2020) 527–542. https://doi.org/10.1093/rb/rbaa042

[8] D.G. O’Shea, C.M. Curtin, F.J. O’Brien, Articulation inspired by nature: a review of biomimetic and biologically active 3D printed scaffolds for cartilage tissue engineering, Biomater. Sci. 10 (2022) 2462–2483. https://doi.org/10.1039/d1bm01540

[9] D.C. Browe, P.J. Díaz-Payno, F.E. Freeman, R. Schipani, R. Burdis, D.P. Ahern, J.M. Nulty, S. Guler, L.D. Randall, C.T. Buckley, P.A.J. Brama, D.J. Kelly, Bilayered extracellular matrix derived scaffolds with anisotropic pore architecture guide tissue organization during osteochondral defect repair, Acta Biomater. 143 (2022) 266–281. https://doi.org/10.1016/j.actbio.2022.03.009

[10] A. Semitela, P.A.A.P. Marques, A. Completo, Strategies to engineer articular cartilage with biomimetic zonal features: a review, Biomater. Sci. 12 (2024) 5961–6005. https://doi.org/10.1039/d4bm00579a

[11] X. Lin, Y. Zhang, J. Li, B.G. Oliver, B. Wang, H. Li, K.T. Yong, J.J. Li, Biomimetic multizonal scaffolds for the reconstruction of zonal articular cartilage in chondral and osteochondral defects, Bioact. Mater. 43 (2024) 510–549. https://doi.org/10.1016/j.bioactmat.2024.10.001

[12] D. Dehghan-Baniani, B. Mehrjou, P.K. Chu, W.Y.W. Lee, H. Wu, Recent advances in functional engineering of articular cartilage zones by polymeric biomaterials mediated with physical, mechanical, and biological/chemical cues, Adv. Healthc. Mater. 12 (2023) e2202581. https://doi.org/10.1002/adhm.202202581

[13] M. Khoshgoftar, W. Wilson, K. Ito, C. van Donkelaar, The effect of tissue-engineered cartilage biomechanical and biochemical properties on its post-implantation mechanical behavior, Biomech. Model. Mechanobiol. 12 (2013) 43–54. https://doi.org/10.1007/s10237-012-0380-0

[14] A.S. Karam, G.S. Kronemberger, K. Chattahy, D. J. Kelly, Cell-Only Bioprinting of Articular Cartilage Progenitor Cells within a Physically Constraining Support Bath to Engineer Structurally Organized Grafts, Bioactive Materials (2025).

[15] G. Dowthwaite, J. Bishop, S. Redman, I. Khan, P. Rooney, D. Evans, L. Haughton, Z. Bayram, S. Boyer, B. Thomson, The surface of articular cartilage contains a progenitor cell population, J. Cell Sci. 117 (2004) 889–897. https://doi.org/10.1242/jcs.00912

[16] S. Patrício, L. Sousa, T. Correia, et al., Freeform 3D printing using a continuous viscoelastic supporting matrix, Biofabrication 12 (2020) 035017. https://doi.org/10.1088/1758-5090/ab8bc3

[17] F.D. Spagnuolo, G.S. Kronemberger, D.J. Kelly, A 4D bioprinting platform to engineer anisotropic musculoskeletal tissues by spatially patterning microtissues into temporally adapting support baths, bioRxiv (2025). https://doi.org/10.1101/2025.05.01.651636
